# Imaging how thermal capillary waves and anisotropic interfacial stiffness shape nanoparticle supracrystals

**DOI:** 10.1038/s41467-020-18363-2

**Published:** 2020-09-11

**Authors:** Zihao Ou, Lehan Yao, Hyosung An, Bonan Shen, Qian Chen

**Affiliations:** 1 Department of Materials Science and Engineering, University of Illinois, Urbana, IL 61801 USA; 2 Materials Research Laboratory, University of Illinois, Urbana, IL 61801 USA; 3 Department of Chemistry, University of Illinois, Urbana, IL 61801 USA; 4 Beckman Institute for Advanced Science and Technology, University of Illinois, Urbana, IL 61801 USA

**Keywords:** Nanoparticles, Self-assembly, Imaging techniques

## Abstract

Development of the surface morphology and shape of crystalline nanostructures governs the functionality of various materials, ranging from phonon transport to biocompatibility. However, the kinetic pathways, following which such development occurs, have been largely unexplored due to the lack of real-space imaging at single particle resolution. Here, we use colloidal nanoparticles assembling into supracrystals as a model system, and pinpoint the key role of surface fluctuation in shaping supracrystals. Utilizing liquid-phase transmission electron microscopy, we map the spatiotemporal surface profiles of supracrystals, which follow a capillary wave theory. Based on this theory, we measure otherwise elusive interfacial properties such as interfacial stiffness and mobility, the former of which demonstrates a remarkable dependence on the exposed facet of the supracrystal. The facet of lower surface energy is favored, consistent with the Wulff construction rule. Our imaging–analysis framework can be applicable to other phenomena, such as electrodeposition, nucleation, and membrane deformation.

## Abstract (web summary)

Interfacial fluctuations at the nanoscale, such as shape evolution of a growing crystal, are prohibitively difficult to study experimentally. Here, the authors are able to map the kinetic and thermodynamic parameters involved in shaping of nanoparticle supracrystals by directly imaging the fluctuating crystal surface by liquid-phase TEM, and analyzing it in the context of capillary wave theory.

## Introduction

The shape and surface morphology of crystalline nanomaterials are of central importance to determine their properties and applications^1–4^. For example, surface roughness alone has been shown to restrict phonon transport below the Casimir limit in crystalline Si nanowires^3^, and to dictate cellular adhesion and proliferation on zirconia nanofilms^4^. When it comes to “supracrystals” assembled non-covalently from nanoparticles (NPs, instead of atoms), the crystal shape and exposed external facets can play a key role in governing their architecture-dependent properties^1,2,5^. For example, supracrystals composed of gold NPs have been shown to exhibit photon–plasmon coupling for potential applications in lasing and quantum electrodynamics^2^, with their photonic modes encoded simply by the supracrystal shape. Numerous theoretical efforts have thus been focused on predicting the shape and surface morphology of crystals (i.e., crystal habit) developed during the growth process over the last few decades^6,7^. Regarding crystal shape, the Wulff construction rule is a prominent principle predicting the thermodynamically stable shape of a crystal by considering the collective effects of the surface energies of different facets of a crystal^7,8^. Extensions of the Wulff construction rule to Winterbottom and Summertop constructions further predict the crystal shapes when the crystal growth is confined by substrates^9^. Regarding surface morphology, a diversity of models on kinetic growth mode such as the Stranski–Krastanov, Frank–van der Merwe, and Volmer–Weber models weigh the balance of factors such as energy landscape of the crystal surface and surface diffusional dynamics, to predict whether building units attach layer-by-layer (relatively smooth) or as islands (relatively rough) to growing crystals^10,11^.

However, despite the broad interest and extensive theoretical models in crystal habit, achieving a high level of control over it at the nanoscale requires addressing a few fundamental gaps. For supracrystals, controlling their surface roughness relies on understanding how NPs attach one by one onto the surface of a growing supracrystal during the self-assembly process in solution, which has been experimentally inaccessible due to the challenge to resolve the motions of individual NPs in solutions at the nanometer resolution^12^. As a result, supracrystal growth dynamics have been largely indirectly inferred from either ex-situ real-space measurements based on electron microscopy images of discrete stages of the growth process in dried samples^13,14^, or from in-situ reciprocal space characterizations like small-angle X-ray scattering^15^. As to controlling supracrystal shapes, the encountered challenges are threefold. All the construction rules (e.g., Wulff, Winterbottom, Summertop) based on the thermodynamic stability require precise surface energy values of different supracrystalline facets as the inputs, which have not been experimentally measured^8,9^. Instead, these values were often estimated by a broken bond model assuming all the interparticle interactions are local, which could be hard to use due to the long-range nature of most colloidal interactions at the nanoscale^16^ or when the building units are highly anisotropic in shape (e.g., rods, prisms, and arrows) and render the interparticle interactions directional. Moreover, NPs can experience high diffusion barriers compared to atoms that these theories were originally established for, so that they might fall into kinetically trapped states which deviate from thermodynamic predictions^17^. Thirdly, simulations of their growth behaviors need to consider the nanoscopic morphology and interaction profiles of the NPs, and the mass and energy transport contributions—surface and volume diffusion, defect annealing, strain relaxation—all at once, which remains challenging and computationally expensive, especially in the absence of experimental data.

Here we utilize a recently developed technique of liquid-phase transmission electron microscopy (TEM) to experimentally map the kinetic and thermodynamic parameters key to crystal growth at the nanoscale, including the kinetic growth profile, facet-dependent interfacial stiffness (the sum of surface energy and its second derivative), surface roughness, and interfacial mobility. Mapping of these otherwise inaccessible parameters is made possible by combining a capillary wave theory (CWT) and the capability to directly image a fluctuating surface of a growing supracrystal at the nanoscale using liquid-phase TEM. Liquid-phase TEM can seal liquid samples against the high vacuum of TEM and capture the dynamics of nanosized entities at the nanometer resolution^18,19^. In this work, we use low-dose liquid-phase TEM imaging to study the shaping process of NP supracrystals during post-nucleation growth. Aided by single-particle tracking, we find that the spatiotemporal fluctuations of the surface profiles of a growing supracrystal match quantitatively with CWT (Supplementary Movie 1), which has been prevalently used for equilibrated interfaces in micron-sized colloid^20,21^ and atomic systems^22–26^. This match is demonstrated here on the single NP level—only accessible with liquid-phase TEM—showcasing the capability of our method to minimize long-standing complications from beam or substrate. From the liquid-phase TEM movies, we capture an intriguing shift in the exposed facets (from (340) to (210)) during the supracrystal growth. Following CWT, we are able to measure the corresponding interfacial stiffness, which pinpoints the energetic driving force for this shift towards lower surface energy facets, leading to a smoother faceted morphology of the supracrystal. This real-space imaging and fluctuation analysis of a growing NP supracrystal provides guidance on encoding the shape and surface morphology of nanostructured materials from the building units and growth kinetics. Our workflow can be potentially extended to other phenomena governed by nanoscale interfacial fluctuations, from substrate guided crystal growth^17^, dendrite formation^27^, fusion of lipid vehicles^28^, to cellular compartmentalization^29^.

## Results

### Fluctuating surface of a NP supracrystal

**Fig. 1:**
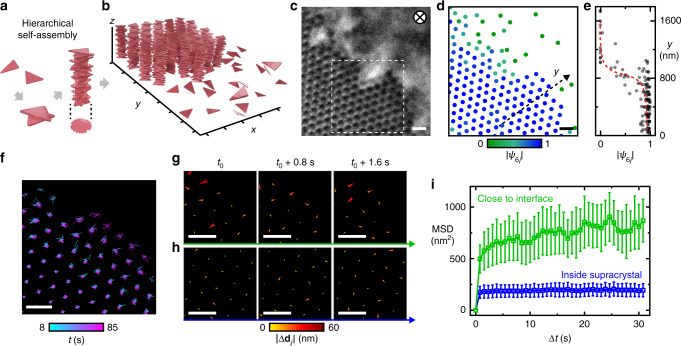
A supracrystal–suspension interface visualized in real-time by liquid-phase transmission electron microscopy (TEM). **a** Schematic of the triangular prisms with favored face-to-face attraction stacking into columns in a misaligned fashion, leading to a circular projection in TEM. **b** Schematic of the supracrystal–suspension interface where the columns assemble laterally into an ordered 3D hexagonal supracrystal. **c** Liquid-phase TEM image of the supracrystal surface interfaced with the suspension of dispersed building blocks. **d** Plot (corresponding to the TEM image in **c**) with single column positions tracked as filled circles, colored according to the *j*th column’s local bond-orientational order parameter $\left( {\left| {\psi _{6j}} \right|} \right)$. **e** Plot of $\left| {\psi _{6j}} \right|$ across the *y* direction labeled in (**d**). Red dashed line is drawn to guide the eye. **f** A trajectory map of columns corresponding to the boxed region in (**c**) measured from time-lapse TEM movies with color coded to elapsed time. **g**, **h** Successive displacement maps of the columns close to the supracrystal–suspension interface (**g**) and inside supracrystal (**h**), where the length and color of the arrows denote |Δ**d**_*j*_|, the magnitude of the displacement. **i** Plot of mean squared displacement (MSD) vs. time Δ*t* of the columns close to the supracrystal–suspension interface (green, mean ± s.e.m. from 18 columns) and inside the supracrystal (blue, mean ± s.e.m. from 12 columns). Scale bars: 200 nm.

In equilibrium, the free surface between two phases is determined by a balance of the flattening effect of surface energy and statistical fluctuations of local interface locations induced by thermal motions. In the framework of CWT, thermally induced capillary waves of different wavelengths superimpose into rough interfacial profiles, where equilibrium roughness is a tradeoff between the surface energy and thermal fluctuation^22,30^. In this context, the three-dimensional (3D) supracrystal system whose nucleation pathway we characterized earlier^31^ under liquid-phase TEM serves as the model system to study nanoscale interfacial fluctuation (Fig. 1, Supplementary Note 1). The surface layer of the supracrystal interfaces with an aqueous suspension of dispersed NPs from which the supracrystal grows. The NP building blocks are gold triangular nanoprisms (100 ± 9.5 nm in side length, 7.5 nm in thickness, Supplementary Fig. 1). They are coated by negatively charged thiols and stay dispersed in deionized water due to an interprism electrostatic repulsion. An ionic strength increase to ~0.5 M screens the interprism electrostatic repulsion to induce the face-to-face stacking of the prisms into cylindrical columns standing vertical to the SiN_*x*_ substrate^31^. As shown in our pairwise interprism interaction calculation (Supplementary Fig. 2), the column adopts the most stable configuration where all of the constituent prisms share the same (*x*, *y*) centroid coordinates as the column. At this configuration, the net attraction energy is huge, of a magnitude larger than 20 *k*_B_*T*. The energy penalty for the two adjacent prisms to deviate from this common centroid position by as small as 10 nm (~10% of the prism side length) is already 2*k*_B_*T*, not favored when agitated only by thermal fluctuations (Supplementary Fig. 2d, Supplementary Note 2). Accordingly, each column can be treated as one cylindrical object fully described by its projected (*x*, *y*) centroid coordinates in our interfacial fluctuation analysis. Variations of the cross-sectional shape of the column do not affect the analysis either because the intercolumn interactions are radially symmetric (Supplementary Note 2). The number of constituent prisms in the columns is estimated to be 20*–*30, resulting in a circularly shaped projection of the columns due to sufficient orientational randomness of the prisms^31^. The columns further order into a large-scale hexagonal lattice, whose boundary is vertical to the view, so that we can track their fluctuation in the *xy* plane at the single particle level over time (Fig. 1a–c, Supplementary Figs. 3–4, Supplementary Note 1–3, Supplementary Movie 2). We use low-dose liquid-phase TEM protocols that our group has developed for NP crystallization studies (dose rates of 3.7–14.8 e^‒^ Å^‒2^ s^‒1^) in a closely-sealed SiN_*x*_ liquid chamber^31,32^. These imaging conditions have been shown to keep the thiols and NPs intact upon electron beam illumination^31,32^. The supracrystal spans several microns in the *xy* plane (Supplementary Fig. 5) and has a clear global six-fold rotational symmetry as suggested by the fast Fourier transform (FFT, inset in Supplementary Fig. 5a).

From the real-space TEM movies, we track the positions of a total of ~220 columns per frame with nanometer resolution (Fig. 1c, d and Supplementary Fig. 6, Supplementary Note 4), from which we confirm the dramatic changes of local structural order and motions of columns across the interface. Quantitatively, the supracrystal region exhibits a high and uniform local structural order as characterized by a six-fold bond-orientational order parameter^33^. This parameter *ψ*_6*j*_ is calculated as $\psi _{6j} = \frac{1}{{Z_j}}\mathop {\sum}\nolimits_{k = 1}^{Z_j} {e^{i6\beta _{jk}}}$ for the *j*th column, in which *Z*_*j*_ is the number of nearest neighbors and *β*_*jk*_ is the angle of the bond linking the *j*th column and its *k*th neighbor. The magnitude of *ψ*_6*j*_ (i.e., |*ψ*_6*j*_|) measures the bond-orientational order on the single column level. Based on this analysis, 92% of the columns inside the supracrystal have |*ψ*_6*j*_| > 0.8 (Supplementary Fig. 5a–c and Supplementary Note 5). The value of |*ψ*_6*j*_| decreases sharply to zero within ~300 nm along the *y* direction (labeled in Fig. 1d) from the supracrystal center to its boundary (Fig. 1e and Supplementary Fig. 5d–f, Supplementary Note 5). As shown in Fig. 1f, the columns close to the supracrystal surface are surrounded by fewer nearest neighbors and exhibit more translational freedom than those in the center of the supracrystal (Fig. 1g, h and Supplementary Fig. 7, Supplementary Note 6, Supplementary Movie 3). For more clarity, we plot the mean-squared displacements (MSD) versus time (Δ*t*) of the columns (Fig. 1i). For the columns in the supracrystal, the MSD reaches a plateau of 200 nm^2^ due to local confinement to the lattice sites, while for the columns near the surface, the MSD increases first and then flattens at ~750 nm^2^, confirming more translational motions due to the less coordinated environment.

### Mapping spatiotemporal fluctuations of surfaces

**Fig. 2:**
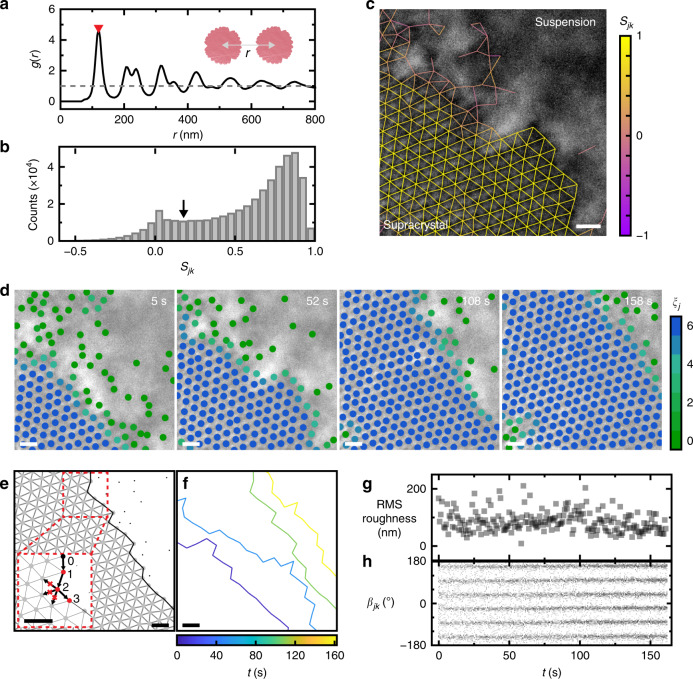
Profiling of the surface layer in the supracrystal system. **a** Radial distribution function *g*(*r*) as a function of *r* (defined in the inset schematic) for the supracrystal computed from Supplementary Movie 2. The red triangle denotes the *r* value corresponding to the lattice constant *σ*. **b** Histogram of the connectivity *S*_*jk*_ computed from Supplementary Movie 2, with *S*_c_ denoted with the black arrow. **c** Liquid-phase TEM image of the supracrystal interfaced with the suspension overlaid with the bond connection network color labeled according to *S*_*jk*_ values. **d** Time-lapse TEM images overlaid with tracked column positions (filled circles colored to the values of *ξ*_*j*_ for the *j*th column). **e** The bond connection diagrams corresponding to one TEM image in **d** (108 s), where columns that belong to the supracrystal are labeled as gray filled circles and others are labeled as black dots. The supracrystal surface, i.e., the outermost surface layer of the supracrystal, is traced as the black line. A zoomed-in view (inset) shows how the columns at the surface are traced one-by-one in sequence. **f** Surface profiles corresponding to the TEM images in **d** color coded by elapsed time. **g** Temporal evolution of the root mean square (RMS) roughness of the surface. **h** Temporal evolution of the bond angles *β*_*jk*_ between the *j*th column and its *k*th nearest neighbor. Scale bars: 200 nm.

At each frame, we delineate the supracrystal surface by determining which columns are connected to each other and evaluating the local order of connected columns. Here the instantaneous profiles of the supracrystal‒suspension interface are defined as the (*x*, *y*) coordinates of the outermost layer of the supracrystal using a local order parameter of the solid bond number (*ξ*_*j*_ for the *j*th column) to differentiate the columns in the supracrystal from those dispersed in the suspension. Solid bond number *ξ*_*j*_ characterizes the number of the nearest-neighbors of the *j*th column that are connected to the *j*th column in a crystalline local environment with similar bond orientations^34,35^. This order parameter serves as a rigorous measure of local crystallinity by eliminating false bond-order coherence originated from dense disordered phase^36,37^. Specifically, the connectivity (*S*_*jk*_) of a bond between the *j*th column and its *k*th nearest-neighbor^35,38^ is computed following $S_{jk} = {\mathrm{Re}}\left( {\psi _{6j}\psi _{6k}^ \ast } \right)$ for a total of 441,704 column pairs in Supplementary Movie 2. The histogram of *S*_*jk*_ shows a characteristic *S*_c_ of 0.175, chosen as the local minimum between the two peaks of the histogram (Fig. 2a, b). Only the bonds of a high connectivity, i.e., *S*_*jk*_ > *S*_c_, are counted as a solid bond in calculating *ξ*_*j*_ (Supplementary Fig. 8 and Supplementary Note 7). As shown in Fig. 2c, there exists a clear distinction in the values of *S*_*jk*_ between the supracrystal and suspension phases. Figure 2d shows the time-lapse liquid-phase TEM snapshots with the *j*th column colored according to *ξ*_*j*_. The columns of *ξ*_*j*_ ≥ 4 are assigned quantitatively as the supracrystal phase. The instantaneous surface profiles are extracted from the coordinates of the columns at the outermost crystalline layer (black lines in Fig. 2e and Supplementary Note 7, Supplementary Movie 2), which are traced by starting from the leftmost column and connecting along the nearest neighboring bond of the smallest bond angle change (Fig. 2e, inset).

Notably, the supracrystal undergoes growth and pushes the surface profile forward over time (Supplementary Fig. 3 and Supplementary Movie 2), yet the roughness of the surface stays constant for a long time, suggesting the fluctuation of the profile is driven by the generic balance between thermal agitation and surface energy. As shown in Fig. 2f, representative surface profiles are plotted with colors that indicate elapsed time. Further analysis of root mean square (RMS) roughness versus time shows a two-stage evolution which we discuss more in detail later: RMS roughness stays constant at (104 ± 36) nm during the first ~100 s and stabilizes to a lower value of (80 ± 25) nm for the later ~50 s (Fig. 2g and Supplementary Fig. 9, Supplementary Note 8). This RMS roughness profile contrasts with the reported power law dependence of RMS roughness versus time when kinetic roughening by random arrival of material on the surface dominates the surface profile^27,39^. We attribute this observation to the time scale separation in the supracrystal growth and surface fluctuation (Supplementary Note 9). Similar time scale separation arguments have been used in systems of micron-sized colloids with ongoing crystal growth^40^ and with grain boundaries deformed under external shear^30^. The orientation of the supracrystal stays unchanged as shown in the bond network (Fig. 2h), facilitating our later analysis on the anisotropy of interfacial stiffness.

### Shift of surface facets during supracrystal growth

For an interface involving a crystalline lattice, surface energy *γ* depends on the direction of the interfacial plane relative to the crystal lattice and is anisotropic because of its structural origin. This anisotropy plays an important role in governing crystal nucleation and shaping crystals into distinctive morphologies^8,41^. Interfacial fluctuations thus depend on both *γ* (related to interfacial stretching) and the second derivative of *γ* with respect to surface orientation (related to interfacial bending), which are better characterized by interfacial stiffness $\tilde \gamma$ (sum of these two contributions^42,43^). Interestingly, in our system, although the lattice orientation of the supracrystal stays unchanged (Fig. 2h), its surface orientation undergoes a shift within our observation time to separate the supracrystal growth process into two stages, due to the very concept of anisotropic interfacial stiffness.

**Fig. 3:**
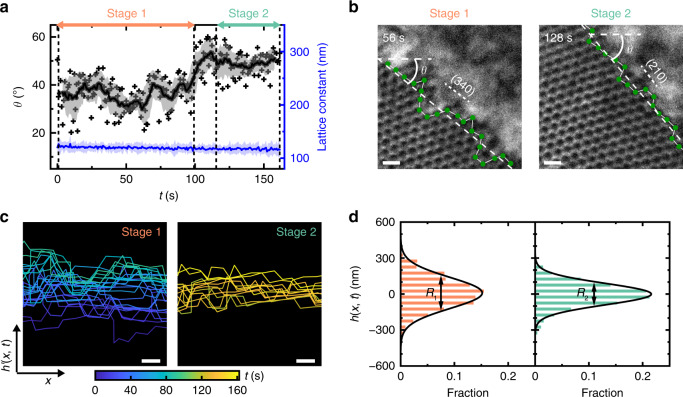
Shift of surface orientation and facet during the supracrystal growth. **a** Plot showing the evolution of the surface orientation (*θ*, black crosses) over time *t* as computed from the surface profiles. The black filled circles are the averaged *θ* over the successive 8.5 s with the gray shadow denoting s.d. Two stages of *θ* are observed and highlighted with salmon and aquamarine arrows. The lattice constant *σ* in the supracrystal (blue line) shows no significant change during growth with the shadow denoting s.d. **b** Typical liquid-phase TEM images for stage 1 and stage 2 highlighting the surface orientation shift. The columns at the surface are labeled as green filled circles connected by white bonds. White dotted lines denote linear regression of the surface profiles. **c** Surface profiles of the supracrystal color coded by elapsed time (4 s interval between neighboring profiles). **d** Corresponding histogram of *h*(*x*, *t*), with the black lines denoting the corresponding Gaussian distributions. The surface roughness *R*_1_ for stage 1 and *R*_2_ for stage 2 are labeled as the full width at half-maximum. Scale bars: 200 nm.

As shown in Fig. 3a, the temporal evolution of surface orientation *θ* exhibits two stages (labeled in salmon and aquamarine), each of which follows its own Gaussian distribution (Supplementary Fig. 9). Here *θ* is defined as the angle between the linear regression of the surface profile^30^ tracked in each TEM snapshot and a horizontal axis (Supplementary Note 10). The representative snapshots for these two stages are shown in Fig. 3b: The surface orientation *θ* undergoes a 19% increase, starting from *θ* = (36 ± 6)° for the first stage of ~100 s, and then shifting to stabilize at *θ* = (49 ± 5)° for the second stage of ~50 s (Supplementary Fig. 9a). The surface orientation dictates the exposed facets (and the growth direction) of the supracrystal, which in our experiment changes from a high index (340) facet to a low index (210) facet, converging to thermodynamically favored lower surface energy state. The orientation shift is also consistent with the notations in atomic crystals that facets of lower Miller indexes tend to have a lower surface energy and be more favored to be exposed at the crystal surface^7^. Despite the shift, the lattice constant *σ* remains the same over the growth process (blue line in Fig. 3a), suggesting that the system is in a stable and nonchanging interaction environment without accumulated beam effects. It is noteworthy that the radiolysis products generated during liquid-phase TEM imaging reach at steady concentrations within seconds^32,44^. At our low dose rates, these products do not react with NPs but lead to an effective ionic strength increase that screens interprism electrostatic interaction, which triggers supracrystal growth^31,32,45^.

The surface of the supracrystal exhibits a lower roughness upon the shift of surface orientation. Typically, the surface roughness is determined by the surface energy existing between two phases against thermal fluctuations^46^. The surface profiles of (*x*, *y*) coordinates depicted in Fig. 3c are defined by both this balancing effect and the supracrystal growth, the latter of which we subtract to measure parameters generic to surface fluctuation (Supplementary Note 10). Specifically, we extract a modified surface profile as a one-dimensional (1D) height function *h*(*x*, *t*) as detailed below. The directly mapped surface profiles are first rotated anti-clockwise by the averaged *θ* of each stage into 1D functions of *h*′(*x*, *t*) (Supplementary Fig. 10a). The increase of *h*′(*x*, *t*) in the direction vertical to the surface is contributed solely by the crystal growth characterized by a constant velocity *v* at each stage, following the literature for crystal growth from micron-sized colloids^40^. As shown in Supplementary Fig. 10b, the growth velocity is 5.30 ± 0.23 nm s^−1^ at stage 1 and increases to 6.52 ± 0.36 nm s^−1^ at stage 2, indicating faster and more favored growth at the later stage. By subtracting the crystal growth contribution, the final obtained height function *h*(*x*, *t*) fluctuates but does not monotonically increase or decrease (Supplementary Fig. 10c, Supplementary Note 10). The accumulated probability distributions of *h*(*x*, *t*) for both two stages fit with a Gaussian distribution centered at *h*(*x*, *t*) = 0 (Fig. 3d), suggesting that the crystal growth contribution is removed, and thermal fluctuations dictate the surface profile^30^. The RMS roughness in each of the two stages (Fig. 2h and Supplementary Fig. 9b) does not exhibit monotonic changes over time, consistently validating no effects from crystal growth.

Both the instantaneous RMS roughness and the average roughness *R* (full width at half maximum of the Gaussian fit for *h*(*x*, *t*)) show that the surface becomes smoother at stage 2 than at stage 1 (Supplementary Movie 4). Stage 1 has an average roughness *R*_1_ of 305 nm, about three times the lattice parameter of the supracrystal (Fig. 3d), while *R*_2_ becomes 31% smaller in stage 2 (210 nm), elucidating that the supracrystal converges towards smooth surfaces during growth, consistent with flat-faceted polygonal shapes of equilibrated supracrystals formed outside TEM^8,13,41,47^ (e.g., cubic, octahedron, rhombic dodecahedron crystals formed by DNA-functionalized gold NPs^41^). Our in-situ TEM imaging sheds light on the dynamic process of the crystal surface development.

### CWT and facet-dependent interfacial stiffness

**Fig. 4:**
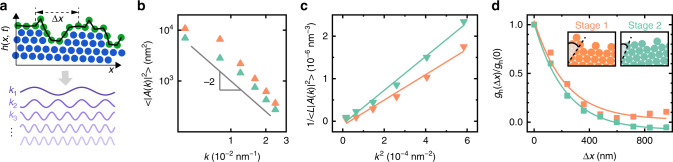
Validation of capillary wave theory (CWT) at the nanoscale and calculation of orientation-dependent interfacial stiffness. **a** Schematic illustrating the Fourier decomposition of the height function *h*(*x*, *t*) into superimposed waves with different wave vectors (*k*_1_, *k*_2_, *k*_3_, …). **b** Dependence of $\langle |A(k)|^2\rangle$ on *k* on a log-log scale plot for stage 1 (salmon) and stage 2 (aquamarine). The gray line of a power law of −2 is drawn to guide the eye. **c** Dependences of $1/L\langle |A(k)|^2\rangle$ on *k*^2^ overlaid with the corresponding linear fitting lines for the two stages. **d** Plot of lateral correlation functions *g*_h_(Δ*x*) normalized by *g*_h_(0) as a function of Δ*x* (defined in **a**) overlaid with exponential fitting for stage 1 (salmon) and stage 2 (aquamarine). Insets highlight the different surface orientations of two stages.

We validate the applicability of CWT to our system, which allows us to extract quantitative measures of anisotropic interfacial stiffness and interfacial mobility. CWT has been successfully applied to simulation studies of atomic interfaces^22^ (e.g., Ni^23,24^, Ag^25^, Fe with different lattice structure^26^) and experimental studies of micron-sized colloidal suspensions^20,21,40,42,48^, but not for a nanoscale fluctuating interface at the single building block resolution. We conduct FFT to decompose *h*(*x*, *t*), only accessible via our direct imaging, into a series of sinusoidal waves with different wave vectors as shown in Fig. 4a. From the equipartition theorem, the time-averaged Fourier coefficient 〈*A*(*k*)〉 for wave vector *k* is expected to follow $\langle |A(k)|^2\rangle = \frac{{k_{\mathrm{B}}T}}{{L\tilde \gamma }}k^{ - 2}$, where $\tilde \gamma$ is the interfacial stiffness, *L* is the length of the supracrystal surface (fixed at 1560 nm), *k*_B_ is the Boltzmann constant, and *T* is the temperature (Supplementary Note 11). Indeed, as we plot the measured 〈|*A*(*k*)|^2^〉 versus *k* (Fig. 4b), we find that it follows a power law of ‒2 as expected from CWT, confirming both the roughness and the height function of the surface are determined by thermal equilibrium.

According to CWT, we calculate the interfacial stiffness $\tilde \gamma$ at the two growth stages, which elucidates an intriguing dependence on surface orientation. Specifically, we plot $1/L\langle |A(k)|^2\rangle$ as a function of *k*^2^, the linear fitting of which measures the interfacial stiffness $\tilde \gamma /k_{\mathrm{B}}T$ as the slope (Fig. 4c). On one hand, the interfacial stiffness values of both the first and second stages are on the order of 10^−14^ J m^−1^, consistent with interface stiffness scaling as ~*k*_B_*T*/*l*_c_, where *l*_c_ is the size of the building block (120 nm in our system). On the other hand, the interfacial stiffness increases from (1.23 ± 0.09) × 10^−14^ J m^−1^ (stage 1) to (1.63 ± 0.08) × 10^−14^ J m^−1^ (stage 2) by 32%, leading to the lower surface roughness as noted above (Fig. 3d). Earlier work has shown that, for a given crystal interfaced with a suspension, the surface orientation with higher interfacial stiffness corresponds to that of lower surface energy^42^. This shift of surface orientation of the growing supracrystal thus effectively lowers the surface energy, matching with the Wulff construction rule to exhibit lowest surface energy facets for an equilibrium crystal shape.

It is noteworthy that molecular dynamics simulations have been mostly used to obtain the surface energy of a NP supracrystal to predict its equilibrium shape based on the various construction rules^8,49^. Our measurements of the orientation-dependent energetic parameters directly from experiments can aid engineering of the surfaces and final morphology of NP supracrystals as well as understanding nanoscale interactions, where discreteness and nonadditivity have made modeling challenging^16,50^. As an example, our CWT-based analysis can reveal a correlation length *ζ* along the supracrystal surface, which measures the characteristic exponential decay length of the lateral static correlation $g_{\mathrm{h}}({\Delta}x) = \langle h(x,t)h(x + {\Delta}x,t)\rangle _{x,t}$. As shown in Fig. 4d, both the data from the first and second stages follow an exponential decay, which agree with the prediction of CWT, exhibiting similar correlation lengths (225 ± 40 nm for stage 1; 224 ± 13 nm for stage 2). These correlation lengths are about two building block size, suggesting a short-ranged intercolumn interaction consistent with our previous results, where the attraction damps to 10% when the distance of the columns is at 25% of column size^31^. We also measure the parameter of interfacial mobility relating the interfacial normal velocity to the driving force (Supplementary Fig. 11 and Supplementary Note 12), which is (0.30 ± 0.16) m^3^ J^‒1^ s^‒1^ for stage 1, consistent with a scaling analysis, suggesting there exists no bulk driving forces other than thermal fluctuation, such as elastic strains, stored energy of deformation, electromagnetic fields or thermochemical gradients^51^.

## Discussion

Using low-dose liquid-phase TEM imaging, single-particle tracking, and quantitative structural analysis, we are able to identify and monitor real-space surface profiles at the nanoscale. Thermal capillary waves are observed with single particle resolution for a NP supracrystal, which allows experimental measurements of a series of otherwise inaccessible physical parameters determining the surface and shape of supracrystals. Impressively, the anisotropic interfacial stiffness we quantify in the experiments shows the growth of supracrystal towards exhibiting flatter surfaces with lower surface energy, consistent with the Wulff construction rule^12,50,52^. Beyond the quasi-equilibrium system we focus on here, where the surface profile is controlled by the balance between surface energy and thermal fluctuations, the imaging of surface profiles can allow mapping of other parameters on other fluctuating systems at the nanoscale. For example, one can measure the bending and stretching modulus in a fluctuating vesicle where inter-lipid interactions add rigidity to the vesicle during vesicle transformation^29^, or measure the deposition laws as the RMS roughness changes with time due to active materials deposition^27,53^. More studies can emerge to use our method to study fluctuations as liquid-phase TEM becomes more compatible with biological samples and out-of-equilibrium field application.

Our capability to measure facet-dependent interfacial stiffness and our demonstration on their role in shaping the supracrystals can advance crystal design at the nanoscale. For example, one can control the interfacial stiffness and crystal habit by utilizing the toolkits of both intrinsic parameters of NP shape and surface chemistry^41,54–56^ as well as extrinsic parameters, such as temperature, pH, and ionic strength^57–59^. Previously reports have shown that changing the length of DNA ligands on the same gold NPs has led to tunability in the lattice symmetry of the interior structure and exposed facets of the supracrystal^17,55,60^. In binary systems, a diversity of supracrystal shapes including the exotic diamond-like lattices has been achieved by introducing two ligand types, two differently sized NPs, or two NP compositions^61^. Our approach can elucidate the fundamental parameter of interfacial stiffness in these systems and make the crystal design predictable. Broadly, complete 2D surface profiles for more complicated supracrystal structures can be possibly achieved by TEM advancements such as SINGLE^62^, ultrafast electron tomography^63^ and focus-based 3D imaging with *z*-slices^64^, which can be analyzed following the protocols presented here. In-situ liquid-phase TEM is also compatible with introducing environmental triggers during the observation^18,65^, to dynamically modify the surface roughness and interfacial stiffness of crystals as one sees the growth.

## Methods

### Chemicals

All chemicals were purchased and used without further purification. Sodium iodide (99.999%, NaI), cetyltrimethylammonium bromide (BioXtra, ≥99%, CTAB), gold (III) chloride trihydrate (≥99.9%, HAuCl_4_), sodium citrate tribasic dihydrate (BioUltra, ≥99.5%), sodium borohydride (99%, NaBH_4_), l-ascorbic acid (BioXtra, ≥99.0%) and sodium hydroxide (99.99%, NaOH) were purchased from Sigma Aldrich. Sodium chloride (99.3%, NaCl, Fisher Scientific), sodium phosphate monobasic monohydrate (99.0–102.0%, NaH_2_PO_4_·H_2_O, EMD Millipore), sodium phosphate dibasic anhydrous (>99%, Na_2_HPO_4_, Acros), and 2-(2-[2-(11-mercapto-undecyloxy)-ethoxy]-ethoxy)-ethoxy-ethoxy-ethoxy-ethoxy-acetic acid (≥95%, HS(CH_2_)_11_(OC_2_H_4_)_6_OCH_2_COOH, Prochimia Surfaces) were purchased and used without further purification. The water used in this work is nanopure water (18.2 MΩ cm at 25 °C) purified by a Milli-Q Advantage A10 system. All glassware used in this work was treated with aqua regia (mixture of HCl and HNO_3_ with a volume ratio of 3:1), fully rinsed with water and dried.

### Synthesis and purification of gold triangular nanoprisms

The gold triangular nanoprisms used in our experiments were synthesized and purified via a seeded growth method according to the literatures^32,45,66–68^. First, a gold NP seed solution was prepared by rapidly mixing an aqueous solution of HAuCl_4_ (250 μL, 10 mM), sodium citrate (500 μL, 10 mM) and ice cold NaBH_4_ (300 μL, 10 mM) sequentially with 18.95 mL water in a 50 mL flask and stirred at 1150 rpm for 1 min. The addition of the NaBH_4_ solution should be fast to obtain small and monodisperse gold seeds. The seed solution was incubated at 40–45 °C for 15 min and cooled down to room temperature. Gold triangular nanoprisms were grown from the gold seeds (usually good within 2 h after preparation). Aqueous solutions of HAuCl_4_ (250 μL, 10 mM), NaOH (50 μL, 100 mM), ascorbic acid (50 μL, 100 mM), and 22 μL of the as-synthesized gold seed solution were sequentially added into 9 mL of 50 mM CTAB solution containing 50 μM NaI in a 20 mL scintillation vial. The solution was hand-shaken for 1 s after each addition and the mixture was left undisturbed for 30 min. The color of the solution gradually changed from colorless to purple, indicating the formation of triangular nanoprisms along with spherical impurities. To purify the nanoprisms, the purple solution was transferred to a 15 mL centrifuge tube and 0.9 mL of 2 M NaCl was added. After the solution was well-mixed, it was left undisturbed for 2 h to induce face-to-face stacking of triangular nanoprisms due to screening of electrostatic repulsion^32^. This solution was centrifuged twice (1st round: 4900 rpm for 30 s; 2nd round: 1350 rpm for 5 s). Immediately after each centrifugation, the supernatant was removed as much as possible using a micropipette. After the 2^nd^ round of centrifugation, several drops of water were first added to the sediments to redisperse the product and 9 mL of 50 mM CTAB was added to keep the prisms stable for long-time storage.

### Surface modification of the gold triangular nanoprisms

Ligand exchange was conducted for the nanoprisms with carboxylate-terminated thiols following a literature method^32,45,69^. The nanoprisms were stored in in 50 mM CTAB solution after synthesis as detailed above. Firstly, two centrifugations were conducted to decrease the concentration of CTAB molecules dissolved in the solution (1st round: 8800 rpm for 8 min; 2nd round: 6600 rpm for 8 min). After the 1st round of centrifugation, the supernatant was removed and the remaining liquid with sediments (~50 µL) was mixed with 8.95 mL of water. After the 2^nd^ round of centrifugation, the supernatant was removed and the remaining liquid with sediments (~50 µL) was mixed with 3.00 mL of water. Secondly, an aqueous solution of thiol molecules (44.26 µL, 7.93 mM) was added to the prism solution and incubated for 30 min. Finally, the solution was sonicated for 5 s and 0.538 mL of 1 M pH = 8 phosphate buffer solution (PBS, composed of 0.07 M NaH_2_PO_4_·H_2_O and 0.93 M Na_2_HPO_4_) was gently added to the solution and left undisturbed overnight. The final solution contained 100 μM of thiol molecules and 0.15 M of pH = 8 PBS, where the PBS solution screens the electrostatic repulsion of deprotonated thiol ligands and to facilitate efficient coating of the gold prism surface. During ~15 h of incubation, the prisms not only were fully covered by thiols but also began to assemble face-to-face stacked and formed into black sediments. Just prior to use for liquid-phase TEM, we diluted this solution to a PBS concentration of 34.5 mM, so that the prisms remained dispersed instead of face-to-face stacked.

### Liquid-phase TEM sample preparation and imaging

Liquid-phase TEM imaging was carried out on a JEOL 2100 Cryo TEM with a spot size 3 with a LaB_6_ emitter at 200 kV using the Protochips Poseidon 210 liquid flow holder. The illumination area is intentionally kept larger than the fluorescent screen of the TEM (~35 cm). The electron beam is sufficiently spread out to minimize artefacts from the boundary and nonuniformity of the illumination area on the supracrystal growth and to achieve sufficiently low dose rates. The movies were captured by a Gatan Ultrascan charge-coupled device (CCD) camera with a 0.1 s exposure time per frame at a rate of 1.3 frames per seconds (fps). In a typical experiment, an aliquot of the nanoprism solution prepared above (34.5 mM pH = 8 PBS buffer solution) was micropipetted on a SiN_*x*_ chip (window: 550 μm × 20 μm, 150 nm spacer flow echip, Protochips), which was then assembled with another SiN_*x*_ chip (window: 550 μm × 20 μm) in a Protochips Poseidon 210 liquid flow TEM holder. The SiN_*x*_ chips were pretreated at a medium RF level for 45 s using a Harrick PDC-23G basic plasma cleaner to render them clean and hydrophilic. During the liquid-phase TEM imaging, the electron dose rates were kept low (3.7–14.8 e^–^ Å^–2^ s^–1^). At this dose rate, the thiol ligands on the nanoprism have been shown to stay intact on the particle surface and remain negatively charged in our previous studies^31,32,45^.

## Supplementary information

Supplementary Information

Peer Review File

Description of Additional Supplementary Files

Supplementary Movie 1

Supplementary Movie 2

Supplementary Movie 3

Supplementary Movie 4

## Data Availability

The data that support the findings of this study are available from the corresponding authors upon request.

## Code availability

Customized Matlab source codes for image processing and particle tracking are freely available at https://github.com/chenlabUIUC/particle-identification, including detailed instruction.
