# Supplementary material for: Imaging how thermal capillary waves and anisotropic interfacial stiffness shape nanoparticle supracrystals
Source: Nat Commun. 2020 Sep 11;11:4555. doi: 10.1038/s41467-020-18363-2 (PMC7486387; doi:10.1038/s41467-020-18363-2)
Supplement: Supplementary file 1 — Supplementary Information [file 41467_2020_18363_MOESM1_ESM.pdf]

Supplementary Information

Imaging How Thermal Capillary Waves and Anisotropic Interfacial Stiffness Shape Nanoparticle Supracrystals

Ou *et al.*

Supplementary Notes

Supplementary Note 1: Self-assembly mechanism of a hexagonal lattice from triangular nanoprisms.

The self-assembly process of the same type of nanoprisms (Supplementary Figure 1), as used here, into a ordered hexagonal lattice has been shown in our previous work[1]. In short, the nanoprisms were suspended in 34.5 mM PBS solution with pH = 8 when first loaded to the liquid chamber for transmission electron microscopy (TEM) imaging. Upon the illumination of electron beam, the effective ionic strength of the illuminated region increased due to radiolysis of water[2,3], which screens the interprism electrostatic repulsion and triggered the self-assembly. Individual prisms were observed to assemble face-to-face into columns due to the highly directional interprism interaction as balanced by van der Waals attraction and electrostatic repulsion. The columns exhibit radially symmetric, weakly attractive intercolumn interactions[1], and further crystallize to a hexagonal supracrystal as shown Supplementary Figure 3.

Supplementary Note 2: Motion and structure of columns composed of stacked nanoprisms.

Each column moves as a whole as illustrated in Supplementary Figure 2a. To explain this observation, we calculated the interaction between a pair of arbitrarily positioned nanoprisms using a previously presented coarse-grained model[1,3-5]. The model is constructed by considering ligand-coated nanoprisms as two parts: a mesh of beads with spacing of Δ = 0.996 nm interacting via van der Waals attraction, representing gold, and a mesh of negatively charged beads (a unit charge of $e = -1.6 \times 10^{-19}$ C for each) with a charge density of -0.048 C m^{-2} covering the basal plane of the nanoprisms, representing the charged carboxylate ligands. The van der Waals attraction u_{vdW} between each pair of gold beads in two prisms was calculated following $u_{\mathrm{vdW}} = -{H\Delta^6}/{\pi^2 {r_{b-b}}^6}$, in which $H = 28.9k_{\mathrm{B}}T$, the Hamaker constant for gold in water, and r_{b-b} is the center-to-center distance between two beads. The electrostatic repulsion u_{el} between each pair of surface charge beads in two prisms was calculated following $u_{\mathrm{el}} = {e^2}/{4\pi\epsilon_0\epsilon_r r_{b-b}}\, e^{-\kappa r_{b-b}}$, where ϵ_0 is the vacuum permittivity, ϵ_r is the relative permittivity of water, and κ is the inverse of Debye length ($\kappa = [2IF^2/(\epsilon_0\epsilon_r RT)]^{1/2}$, I: ionic strength; F: Faraday constant; R: gas constant; T: temperature). The total pairwise interaction energy is the summation over all interactions between beads of the two nanoprisms. Following this model, the energy penalty for the nanoprisms to slide laterally away from the column center is high, about $2k_{\mathrm{B}}T$ for 10 nm deviation from the center along all the different orientations and sliding directions as shown in Supplementary Figure 2 b–d.

Based on the geometry of the liquid chamber and Monte Carlo simulation shown in our previous work[1], the number of nanoprisms in one column is estimated to be 20–30, resulting in a circularly shaped projection of the columns. Due to the randomness in the nanoprism orientation, inter-column interaction is isotropic in *x-y* plane and columns interact as nearly "hard" cylinders with an attraction of $0.3k_{\mathrm{B}}T$ at the experimental ionic strength I = 0.5 M.

Supplementary Note 3: Image processing protocol for liquid-phase TEM images.

The liquid-phase TEM images in Fig. 1c, Fig. 2c, and Fig. 3b were processed following the procedures specified in Supplementary Figure 4. From the raw liquid-phase TEM images, background subtraction (rolling ball radius: 200 pixels) and contrast enhancement (saturated pixels: 0.3%) were applied in ImageJ[6]. For the clear presentation of tracked column as filled colored circles in Fig. 2d, the grayscale colormap of the contrast enhanced image was modified as shown in Supplementary Figure 4d.

Supplementary Note 4: Automatic tracking at the single column level.

A customized Matlab code was used to track the centroid positions of columns in the liquid-phase TEM movies as detailed in Supplementary Figure 6 using one TEM image in Supplementary Movie 2 as an example (code available at https://github.com/chenlabUIUC/particle-identification). First, a band-pass filter from literature[7] was applied to the TEM image. Parameters used in the function for spatial wavelength

cutoff are 3 and 25 pixels. Second, an intensity threshold was applied to clean pixels with intensity values lower than 15. Third, using the built-in Matlab circle-finding function (imfindcircles.m, with threshold, sensitivity, and radius range set to 0.03, 0.9, and 6–15 pixels, respectively), the position of each column was tracked from the processed image as the centroids of the fitted circles. Occasionally, two tracked "columns" can have a center-to-center distance r smaller than the column size, as shown in the first unphysical peak in the raw radial distribution function (Supplementary Figure 6e). This tracking error occurs when two circles were fitted to an ellipsoidal shape due to motion blurring. Corrections to the column positions were thus applied when r between any two columns was closer than 70.2 nm (dotted line in Supplementary Figure 6e, the first minimum after the unphysical peak in the raw radial distribution function) to only keep one of them. The column positions (x_j, y_j) were obtained and labeled in Figs. 1d, 2d, and Supplementary Movie 2. Parameters in this protocol were slightly modified in the 1st and 3rd steps to optimize the column positions tracked for an ordered hexagonal lattice as shown in Supplementary Figure 5b.

Supplementary Note 5: Structure characterization of the hexagonal supracrystal.

Based on the single column positions (x_j, y_j) extracted following procedures detailed in Supplementary Note 4, the radial distribution function $g(r)$ of the columns were calculated following a literature method[8] as shown in Fig. 2a. The first minimum in $g(r)$, r_c = 170 nm, was used to determine a nearest neighboring bond and the number of nearest neighbors Z_j of the jth column. A local six-fold bond orientational order parameter was calculated for each column following $\psi_{6j} = \frac{1}{Z_j}\sum_{k=1}^{Z_j} e^{i6\beta_{jk}}$, where β_{jk} is the angle of the bond linking particle j and its kth neighbor[9], from which we computed the bond angle plot shown in Fig. 2h and the histogram of $|\psi_{6j}|$ in Supplementary Figure 5c. The value of $|\psi_{6j}|$ changes rapidly across the interface as shown in Fig. 1e, which was obtained by rotating the TEM image to make y direction labeled in Fig. 1d as the vertical axis (Supplementary Figure 5d–f).

Supplementary Note 6: Calculation of the displacement and trajectory maps in Fig. 1f–h.

The trajectory and displacement maps were calculated based on the column positions (x_j, y_j) extracted following procedures detailed in Supplementary Note 4. A trajectory linking algorithm from the literature[7] was applied to identify the temporal trajectory of each column (input parameters: no missing points, maximum displacement 52 nm). The displacement vector $\mathbf{d}_j(t)$ for the jth column from time t to $(t + \Delta t)$ was defined as $\mathbf{d}_j(t, \Delta t) = \left(x_j(t + \Delta t) - x_j(t), y_j(t + \Delta t) - y_j(t)\right)$ as shown in Supplementary Figure 7. Here Δt was chosen as 1/1.3 s, the time interval between adjacent frames. In Fig. 1f–h, drift correction was applied by calculating and then subtracting an averaged trajectory from the displacement of columns in the left-bottom corner of the view in Fig. 1f.

Supplementary Note 7: Identification of the supracrystal surface.

The profile of supracrystal surface, i.e., the supracrystal–suspension interface, is defined as the coordinates of the outermost layer of the supracrystal (Fig. 2e and Supplementary Figure 8). Following previous studies on phase transitions[1,10-12], solid bond number ξ_j was used to characterize how many neighbors belonging to a similar solid-like structure for the jth column. To obtain ξ_j, we computed the connectivity $S_{jk} = \mathrm{Re}\left(\psi_{6j}\psi_{6k}^*\right)$, following previous literatures on crystals formed from micron-sized colloids[13]. We computed S_{jk} for each bond connecting the jth column and its kth nearest neighbor (Fig. 2c, Supplementary Figure 8b), from the distribution of which we extracted a threshold value S_c = 0.175 to determine whether two connected neighbors belong to a similar structure. The parameter ξ_j was calculated as $\xi_j = \sum_{k=1}^{Z_j} \mathrm{H}\left(S_{jk} - S_c\right)$, where H is the Heaviside function[10]. From the local mapping of ξ_j given in Fig.

2d and Supplementary Figure 8c, an interface was observed that separates the supracrystal from the suspension. Quantitatively, we assigned the columns with $\xi_j \geq 4$ as belonging to the supracrystal[1].

Supplementary Note 8: Instantaneous root mean square (RMS) roughness (Fig. 2g).

We used RMS roughness[14,15] to describe instantaneous roughness of a surface. From the surface profile extracted (Supplementary Note 7), a linear regression was applied to extract the orientation of the surface and the image was rotated to make the surface orientation as the x axis (Supplementary Figure 5d–e). Column positions at the surface were then presented as (x_j, y_j), which are discrete points describing the contour of the surface at time t. Linear interpolation was then applied to make a continuous contour (x, y) and the maximum y was kept when there are multiple y values at one x position following a literature convention[15]. The average surface position at time t is then defined as: $\bar{y}(t) = (\sum_x y(x,t))/N$, where N is the number of pixels corresponding to an surface length L of 1560 nm, fixed to eliminate the influence of L on RMS roughness[16]. RMS roughness is further defined as: $((\sum_x y^2(x,t))/N - \bar{y}(t)^2)^{\frac{1}{2}}$. The temporal evolution of RMS roughness is shown in Fig. 2g and the accumulated distributions are given in Supplementary Figure 9b.

Supplementary Note 9: Scaling analysis of physical quantities related to surface.

The estimated characteristic surface fluctuation time scale τ, namely the capillary time scale[17], was calculated based on $\tau = \sigma\zeta\eta/\tilde{\gamma}$ (for a 2D system such as ours), to be about 0.7 ms, where σ is the lattice constant (120 nm), ζ is the correlation length measured from Fig. 4d (225 nm for stage 1), η is the dynamic viscosity of solution (here we used the viscosity of water at 25 °C, 8.9×10^{-4} Pa s), and $\tilde{\gamma}$ is the 2D interfacial stiffness of the supracrystal–suspension interface (different from the interfacial tension in the liquid–liquid interface). It is expected that interfacial tension γ scales as $\sim k_B T/\sigma$ in 2D system[18]. We used an interfacial stiffness value $\tilde{\gamma}$ of 3.4×10^{-14} J m^{-1} in the estimation of the capillary time scale.

Typical interfacial stiffness values reported in 2D atomic simulations are on the order of 10^{-11} J m^{-1}, in which the building block size[19] is on the scale of 10^{-10} m. In model system of micron-sized colloids with size ~1 μm, the 2D interfacial stiffness values[20] have been reported to be on the order of $\sim10^{-15}$ J m^{-1}. These values are consistent with the stiffness scaling[20] as $\sim k_B T/l_c$ in 2D (would be $k_B T/{l_c}^2$ for a 3D system), in which l_c is the size of building block in the system, k_B is the Boltzmann constant, and T is temperature. In our system, the lattice constant σ is 120 nm (Fig. 2a). The expected interfacial stiffness based on the scaling analysis is 3.4×10^{-14} J m^{-1}, which is on the same order of magnitude as those measured in our experiment. Similarly, interfacial mobility can be estimated using scaling analysis as $\sim {l_c}^3/(k_B T t_c)$ in a 2D system, in which t_c is the characteristic time scale. Here, we used the experiment observation time scale (~1 s), which gives an estimated interfacial mobility of 0.4 m^3 J^{-1} s^{-1}, comparable to the value of 0.30 ± 0.16 m^3 J^{-1} s^{-1} measured in our experiment based on capillary wave theory (CWT) (Supplementary Note 12).

Supplementary Note 10: Surface orientation and height function of the supracrystal–suspension interface.

The instantaneous surface orientation θ was measured based on the linear regression of the surface profile[21] obtained above, namely the angle of the fitting line to a horizontal axis. The temporal evolution of θ divides itself into two stages, whose histograms follow Gaussian distributions as shown in Supplementary Figure 9a. Next all the surface profiles in stage 1 were rotated 36° (49° for stage 2) anticlockwise so that the they were parallel to the x axis. Thus, the surface profiles can be written as a one-dimensional (1D) raw height function $h'(x,t)$ (Supplementary Figure 10a). The supracrystal growth velocity v at each stage was measured from the slope of the linear fitting of the $\langle h'(x,t)\rangle - t$ curve, where h_0 was the intercept at x = 0 (Supplementary Figure 10b). Finally, the height function $h(x,t)$ was calculated with the growth effect subtracted following $h(x,t) = h'(x,t) - vt - h_0$ (Supplementary Figure 10c).

Supplementary Note 11: Interfacial stiffness measured based on CWT (Fig. 4).

The interfacial stiffness was calculated from the height function $h(x,t)$ based on CWT[17,22,23]. As $h(x,t)$ is a discrete function of column positions at the supracrystal–suspension interface, a linear interpolation was first conducted to generate a continuous surface profile for fast Fourier transform (FFT). For the FFT calculation, we kept the supracrystal surface of a length L of 1560 nm to eliminate changes in the wave vector. As a result, the minimum wave vector was 4.0×10^{-4} nm^{-1} ($k = 2\pi/L$) due to the finite surface length. The maximum wave vector was selected to be 2.4×10^{-2} nm^{-1}, corresponding to a real-space wavelength of 260 nm, larger than the correlation length ζ (Fig. 4d) of the system.

Supplementary Note 12: Validation of CWT and calculation of interfacial mobility of the fluctuating surface.

In our system, CWT applies although the supracrystal underwent continuous growth and did not reach at equilibrium. Similarly, previous studies on micron-sized colloids also applied CWT to growing colloidal crystals[22] and grain boundaries under external shear[20,21]. We attribute the consistency with CWT in our system to the separation in the crystal growth time scale and the characteristic surface fluctuation time scale (Supplementary Note 9). Specifically, the time required for growing one layer of supracrystal is ~20 s for stage 1, ~16 s for stage 2 based on the growth velocity measured in Supplementary Figure 10b. As to the characteristic surface fluctuation time scale, we studied the time needed for the surface profiles to become fully randomized. In particular, we monitored the temporal trajectory $h(x_0,t)$ of the surface height at a fixed position of $x = x_0$ (Supplementary Figure 11b). Clearly $h(x_0,t)$ fluctuates stochastically at a magnitude of ~150 nm, consistent with the ensemble distribution of $h(x,t)$ in Fig. 3d, showing that there is no temporal correlation between neighboring frames (0.8 s) at each position along the surface.

Temporal correlation between the height function $h(x,t)$ was calculated from the dynamic (temporal) correlation function $g_{\mathrm{h}}(\Delta t) = \langle h(x,t)h(x,t+\Delta t)\rangle_{x,t}$. The dynamic correlation function $g_{\mathrm{h}}(\Delta t)$ characterizes the time it takes for a surface to evolve into a unrelated profile as shown in the schematic of Supplementary Figure 11a. Capillary wave theory predicts that dynamic correlation function follows $g_{\mathrm{h}}(\Delta t) = (k_{\mathrm{B}}T\zeta/\tilde{\gamma}) \times eftc((\Delta t/\tau_0)^{1/2})$, in which $eftc$ is the error function, ζ is the correlation length, $\tilde{\gamma}$ is the interfacial stiffness and τ_0 is the correlation time that we obtained from the fitting[17,20]. The calculated dynamic correlation function for stage 1 is presented in Supplementary Figure 11c and the correlation time measured is 13.9 ± 1.5 s. Interfacial mobility M was then calculated following: $M = \zeta^2/(\tilde{\gamma}\tau_0)$ and the mobility measured is 0.30 ± 0.16 m^3 J^{-1} s^{-1}.

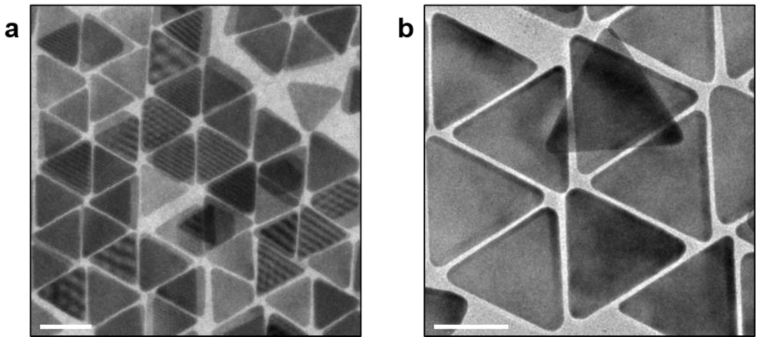

Supplementary Figure 1. Dry transmission electron microscopy (TEM) images of gold triangular nanoprisms. **a** Low-magnification TEM image showing the overall sample quality. Scale bar: 100 nm. **b** High-magnification TEM image showing the detailed morphology of the prisms. Scale bar: 50 nm.

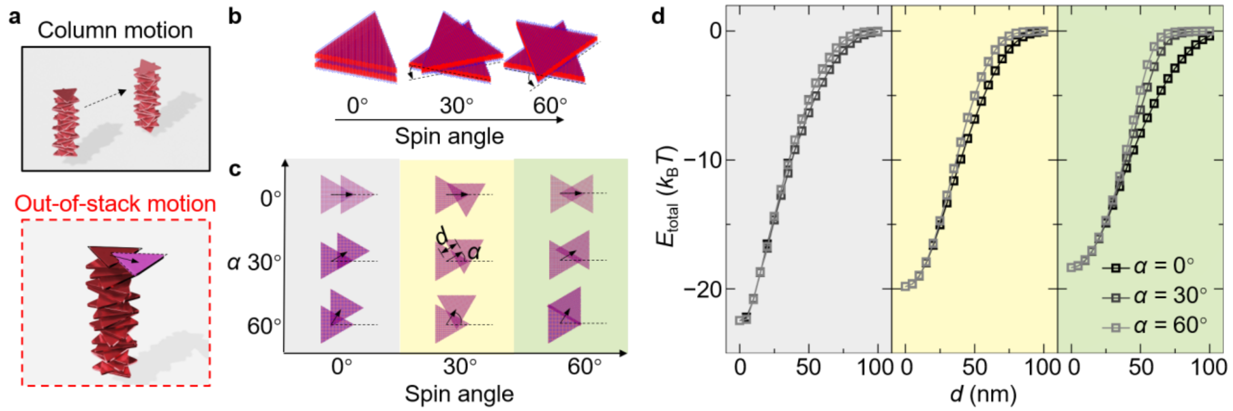

Supplementary Figure 2. Pairwise interaction calculation illustrating the prohibited out-of-stack motion of single nanoprism. a Schematic showing the column moving together as a whole after self-assembly (solid black box) and prohibited out-of-stack motion of nanoprisms (dashed red box). **b** Calculation model showing a pair of stacked nanoprism with different spin angles. The model considers the van der Waals attraction between gold atoms (red) and electrostatic repulsion between negatively charged surface ligands (blue). **c** Configurations of the prism pair with different spin angles and moving directions (α). **d** Graph showing how the computed pair-wise interaction changes as a function of moving distance (d) with different spin angles and moving directions. The results are calculated at ionic strength I = 0.5 M with a vertical separation between two nanoprisms fixed at 14.5 nm. Different shading colors correspond to different spin angles showing in **c**.

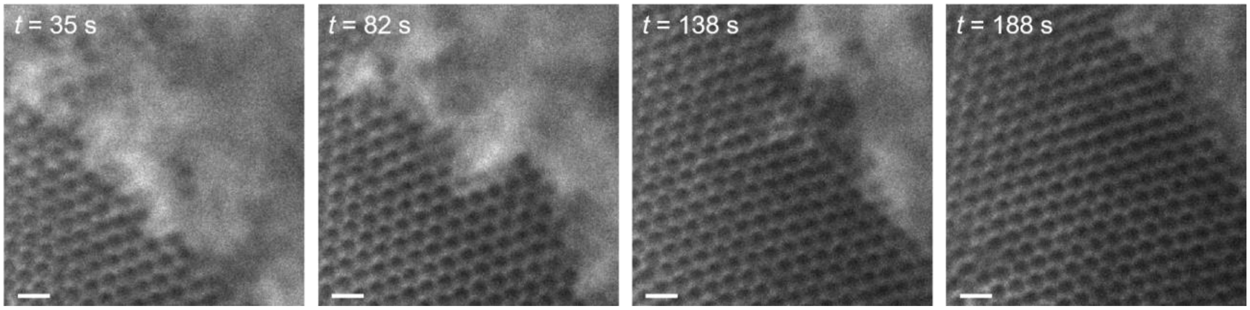

Supplementary Figure 3. Raw time-lapse TEM image series showing the expansion and fluctuation of the supracrystal–suspension interface of those presented in Fig. 2d. Scale bars: 200 nm.

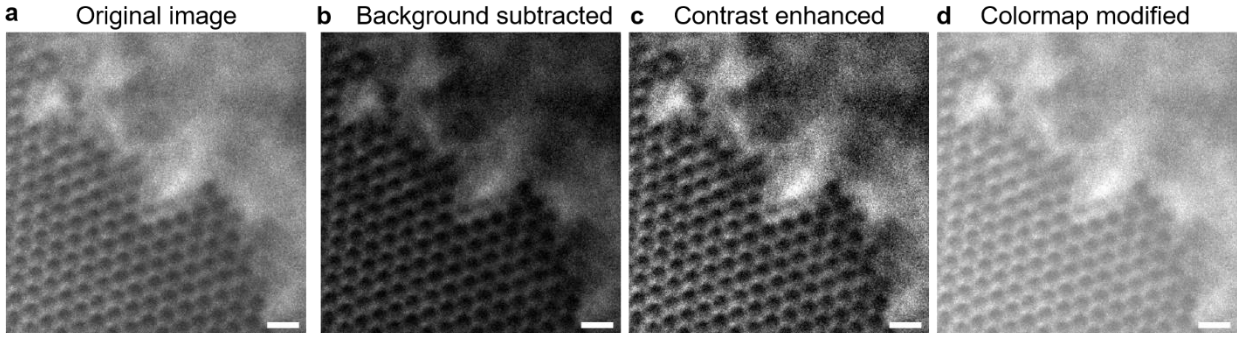

Supplementary Figure 4. Typical processing workflow for liquid-phase TEM images. a–d Step-by-step image processing from the raw liquid-phase TEM image (**a**), the image after background subtraction (**b**), the image after applying contrast enhancement (**c**), to the image after rescaling the grayscale colormap (**d**). Scale bars: 200 nm.

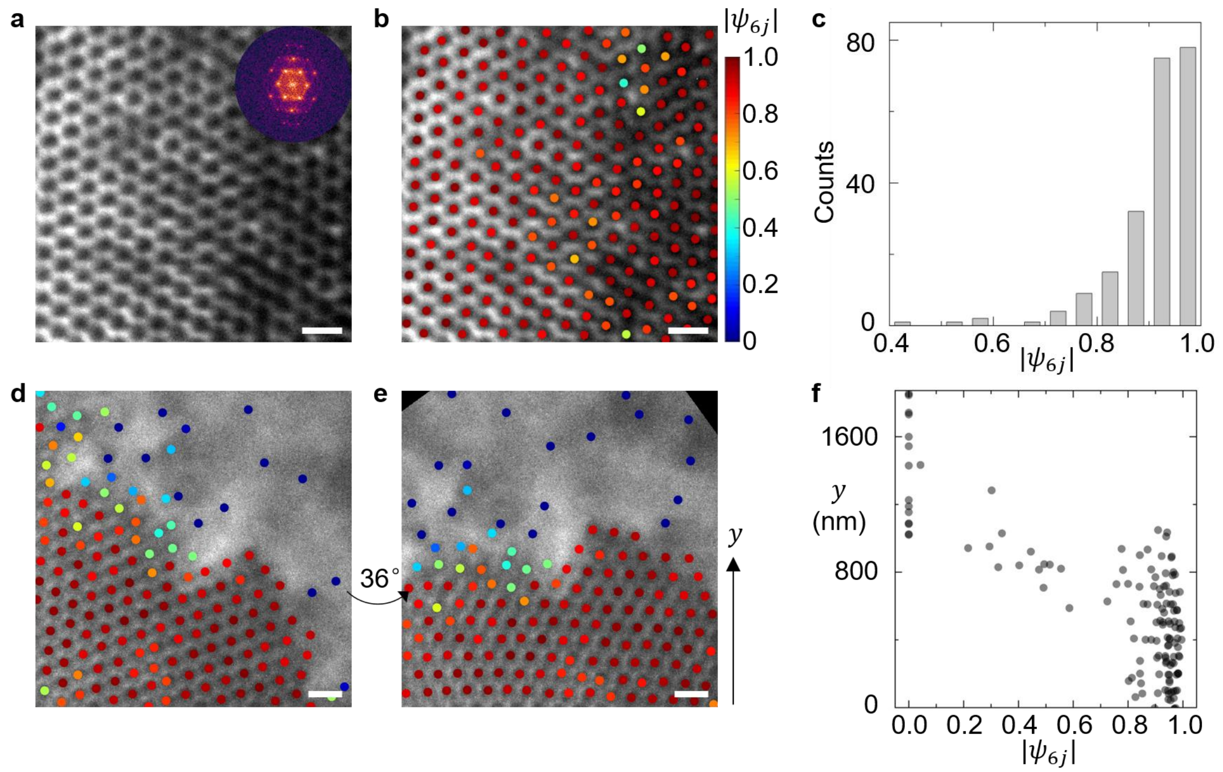

Supplementary Figure 5. Local hexagonal structural order in the supracrystal and across the supracrystal–suspension interface. a Liquid-phase TEM image of the supracrystal. The inset fast Fourier transform (FFT) shows the single crystallinity. **b** The TEM image overlaid with automatically tracked single column positions (filled circles) with colors denoting the magnitude of the local bond orientational order parameter $|\psi_{6j}|$. **c** Histogram of the $|\psi_{6j}|$ values for all the columns in **a**. **d** Liquid-phase TEM image of the supracrystal–suspension interface overlaid with single column positions (filled circles) with colors denoting $|\psi_{6j}|$. **e** The rotated image from **d** to make the interface orientation as the horizontal axis. **f** Dependence of $|\psi_{6j}|$ on y coordinates (y axis labeled in **e**). **b**, **d**, and **e** share the same color map. Scale bars: 200 nm.

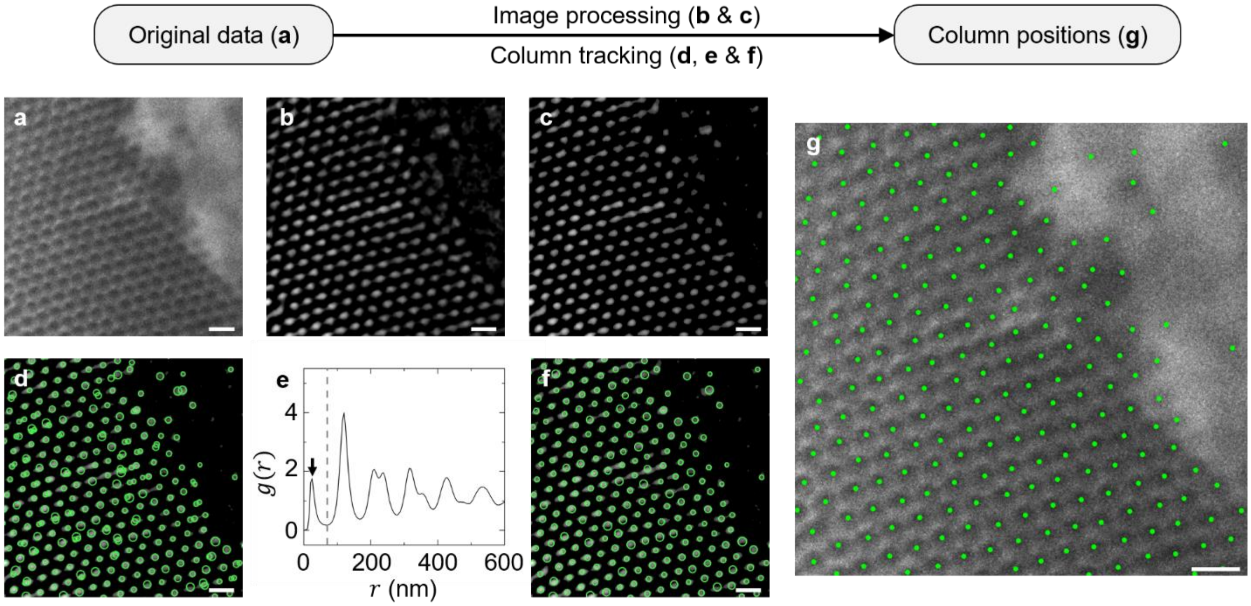

Supplementary Figure 6. Automatic tracking of the centroid positions of columns. Top: Workflow showing the tracking process from a raw liquid-phase TEM image (108 s in Fig. 2d and Supplementary Movie 2). **a** Representative raw liquid-phase TEM image including the supracrystal–suspension interface. **b** The processed image after applying a band-pass filter to **a**. **c** The processed TEM image after applying an intensity threshold filter onto **b**. **d** The processed image (**c**) overlaid with columns tracked as the circular features (green open circles). **e** The raw radial distribution function $g(r)$ calculated from the centroids of the circles in **d.** The arrow marks the unphysical peak and the dashed line denotes the position of the first minimum of $g(r)$. **f** The processed TEM image (**c**) overlaid with columns tracked after eliminating the duplicated centroids tracked from one column. **g** Raw TEM image overlaid with positions of each column tracked (centroid positions of circles in **f**). Scale bars: 200 nm.

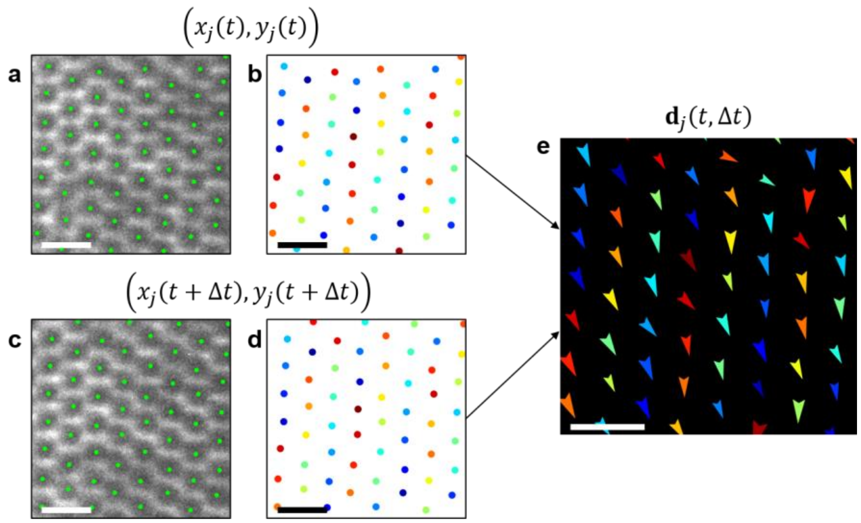

Supplementary Figure 7. Displacement map calculation. a, **c** Liquid-phase TEM images with positions of columns overlaid as green dots at two different time: t and $t + \Delta t$. **b**, **d** Positions of columns at time t and $t + \Delta t$, respectively, with color denoting the column label j. **e** Displacement map calculated from the positions given in **a**, **c**. The colors of arrows follow the column label j in **b**, **d** and the arrow size is proportional to the magnitude of displacements. Scale bars: 200 nm.

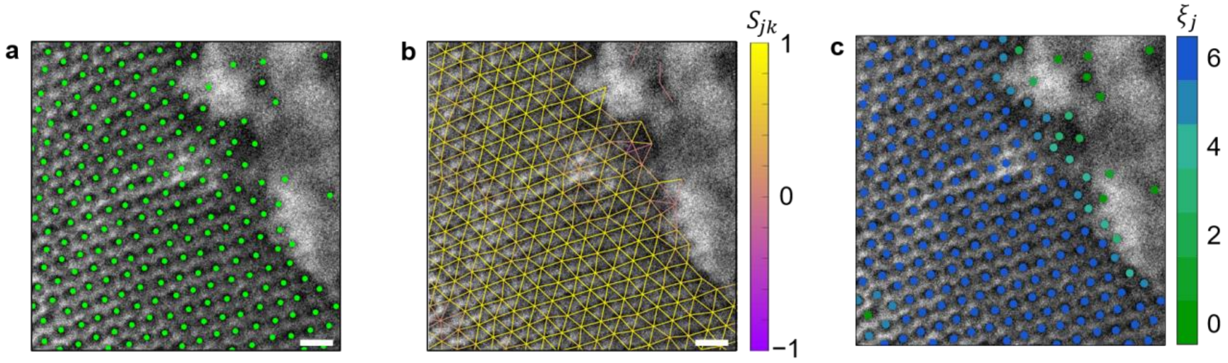

Supplementary Figure 8. Identification of the supracrystal–suspension interface. a Liquid-phase TEM image overlaid with tracked column positions (green dots). **b** The same TEM image in **a** overlaid with bond network colored by the value of S_{jk}. **c** The same TEM image in **a** overlaid with dots colored by the solid bond number ξ_j (108 s, shown in Fig. 2d). Scale bars: 200 nm.

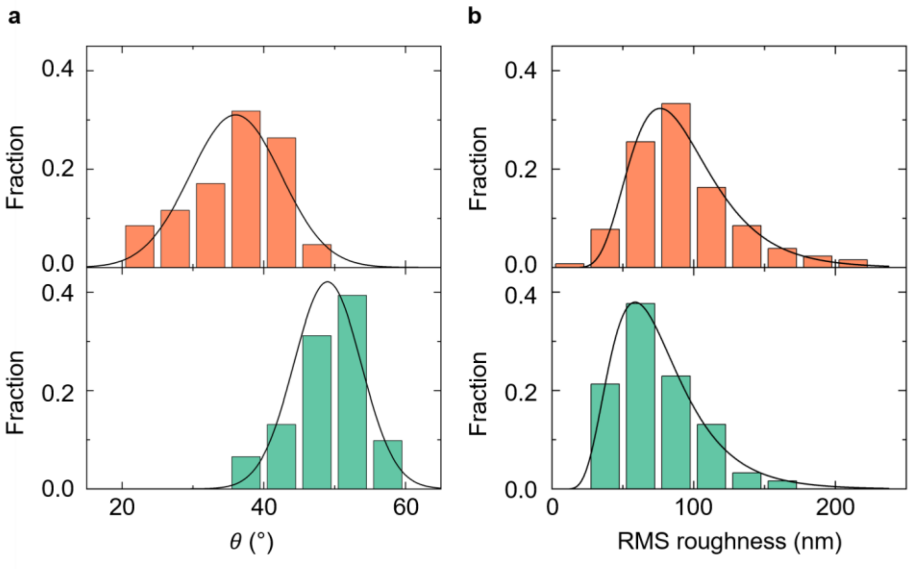

Supplementary Figure 9. Histograms of surface orientation θ and root mean square (RMS) roughness for stage 1 (salmon) and stage 2 (aquamarine). a Histograms of surface orientation θ. Gaussian distributions were used to fit the histograms (solid line) for both stages. **b** Histograms of RMS roughness. Lognormal distributions were used to fit the histograms (solid line) for both stages.

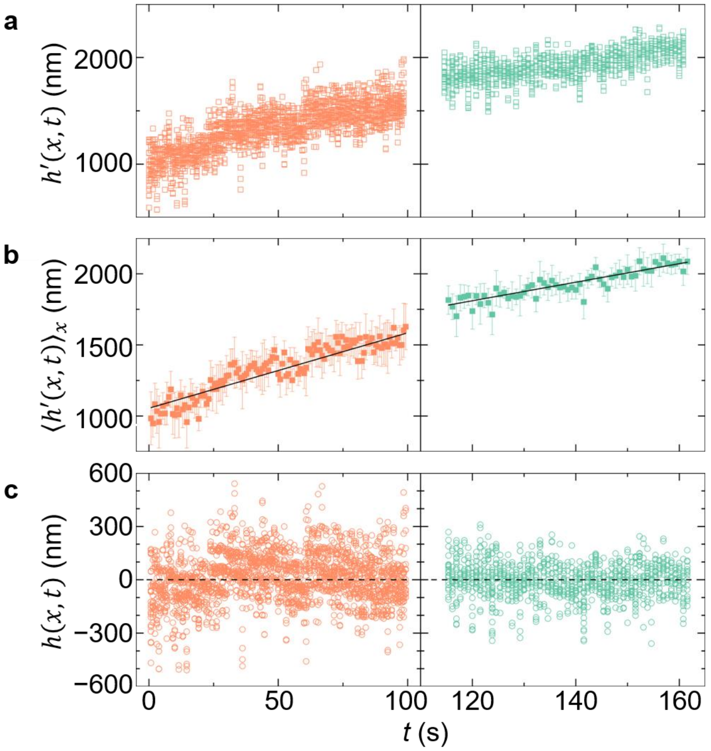

Supplementary Figure 10. Temporal evolution of height functions for stage 1 (salmon) and stage 2 (aquamarine). a Plots showing the temporal evolution of $h'(x,t)$ for the two stages. **b** Plot showing the temporal evolution of the averaged $\langle h'(x,t)\rangle$ over time t for the two stages (mean ± s.d. calculated from $h'(x,t)$ for each time). Black lines are the linear fitting of $\langle h'(x,t)\rangle$ versus t, the slopes of which are growth velocities v of the supracrystal. **c** Plots showing the temporal evolution of $h(x,t)$ for the two stages. Dashed line indicates $h(x,t) = 0$ and the corresponding histograms of $h(x,t)$ for the two stages are shown in Fig. 3d.

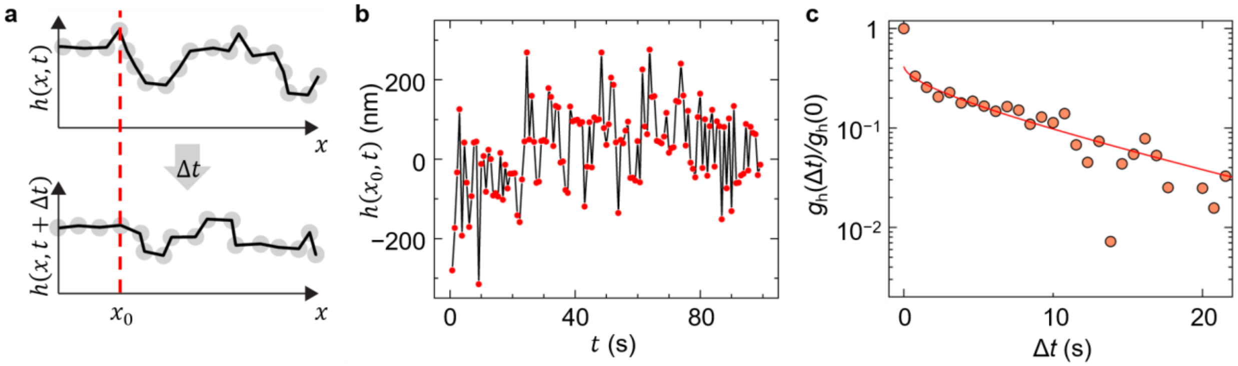

Supplementary Figure 11. Temporal trajectory of a single surface point. a Schematic showing how the height function $h(x,t)$ evolve to $h(x,t+\Delta t)$ during a time gap of Δt. The fixed point with $x = x_0$ is marked with red dotted line. **b** Trajectory of a single surface point ($x = x_0$) in stage 1. **c** Normalized dynamic correlation function $g_{\mathrm{h}}(\Delta t)/g_{\mathrm{h}}(0)$ for stage 1. Red solid line is the fitting lines using function $g_{\mathrm{h}}(\Delta t)/g_{\mathrm{h}}(0) = C \times eftc\left((\Delta t/\tau_0)^{1/2}\right)$, in which $eftc$ is the error function, C is a constant, and τ_0 is the correlation time we are able to measure from the fitting.

Supplementary References:

1. Ou, Z., Wang, Z., Luo, B., Luijten, E. & Chen, Q. Kinetic pathways of crystallization at the nanoscale. *Nat. Mater.* **19**, 450–455 (2020).
2. Kim, J., Jones, M. R., Ou, Z. & Chen, Q. In situ electron microscopy imaging and quantitative structural modulation of nanoparticle superlattices. *ACS Nano* **10**, 9801–9808 (2016).
3. Kim, J., Ou, Z., Jones, M. R., Song, X. & Chen, Q. Imaging the polymerization of multivalent nanoparticles in solution. *Nat. Commun.* **8**, 761 (2017).
4. Gao, B., Arya, G. & Tao, A. R. Self-orienting nanocubes for the assembly of plasmonic nanojunctions. *Nat. Nanotech.* **7**, 433–437 (2012).
5. Luo, B., Kim, A., Smith, J. W., Ou, Z., Wu, Z., Kim, J. & Chen, Q. Hierarchical self-assembly of 3D lattices from polydisperse anisometric colloids. *Nat. Commun.* **10**, 1815 (2019).
6. Schindelin, J., Arganda-Carreras, I., Frise, E., Kaynig, V., Longair, M., Pietzsch, T., Preibisch, S., Rueden, C., Saalfeld, S., Schmid, B., Tinevez, J.-Y., White, D. J., Hartenstein, V., Eliceiri, K., Tomancak, P. & Cardona, A. Fiji: an open-source platform for biological-image analysis. *Nat. Methods* **9**, 676–682 (2012).
7. Crocker, J. C. & Grier, D. G. Methods of digital video microscopy for colloidal studies. *J. Colloid Interface Sci.* **179**, 298–310 (1996).
8. Frenkel, D. & Smit, B. *Understanding Molecular Simulation: from Algorithms to Applications* (Academic press, 2001).
9. Savage, J. R., Blair, D. W., Levine, A. J., Guyer, R. A. & Dinsmore, A. D. Imaging the sublimation dynamics of colloidal crystallites. *Science* **314**, 795–798 (2006).
10. Tan, P., Xu, N. & Xu, L. Visualizing kinetic pathways of homogeneous nucleation in colloidal crystallization. *Nat. Phys.* **10**, 73–79 (2013).
11. ten Wolde, P. R., Ruiz-Montero, M. J. & Frenkel, D. Simulation of homogeneous crystal nucleation close to coexistence. *Faraday Discuss.* **104**, 93–110 (1996).
12. ten Wolde, P. R., Ruiz-Montero, M. J. & Frenkel, D. Numerical calculation of the rate of crystal nucleation in a Lennard-Jones system at moderate undercooling. *J. Chem. Phys.* **104**, 9932–9947 (1996).
13. Tang, X., Rupp, B., Yang, Y., Edwards, T. D., Grover, M. A. & Bevan, M. A. Optimal feedback controlled assembly of perfect crystals. *ACS Nano* **10**, 6791–6798 (2016).
14. Schneider, N. M., Park, J. H., Grogan, J. M., Steingart, D. A., Bau, H. H. & Ross, F. M. Nanoscale evolution of interface morphology during electrodeposition. *Nat. Commun.* **8**, 2174 (2017).
15. Family, F. Dynamic scaling and phase transitions in interface growth. *Physica A* **168**, 561–580 (1990).
16. Takeuchi, K. A., Sano, M., Sasamoto, T. & Spohn, H. Growing interfaces uncover universal fluctuations behind scale invariance. *Sci. Rep.* **1**, 34 (2011).
17. Aarts, D. G. A. L., Schmidt, M. & Lekkerkerker, H. N. W. Direct visual observation of thermal capillary waves. *Science* **304**, 847–850 (2004).
18. Savage, J. R. & Dinsmore, A. D. Experimental evidence for two-step nucleation in colloidal crystallization. *Phys. Rev. Lett.* **102**, 198302 (2009).
19. Trautt, Z. T. & Upmanyu, M. Direct two-dimensional calculations of grain boundary stiffness. *Scr. Mater.* **52**, 1175–1179 (2005).
20. Skinner, T. O., Aarts, D. G. & Dullens, R. P. Grain-boundary fluctuations in two-dimensional colloidal crystals. *Phys. Rev. Lett.* **105**, 168301 (2010).
21. Gokhale, S., Nagamanasa, K. H., Santhosh, V., Sood, A. K. & Ganapathy, R. Directional grain growth from anisotropic kinetic roughening of grain boundaries in sheared colloidal crystals. *Proc. Natl. Acad. Sci. U.S.A.* **109**, 20314–20319 (2012).

22. Nguyen, V. D., Hu, Z. B. & Schall, P. Single crystal growth and anisotropic crystal–fluid interfacial free energy in soft colloidal systems. *Phys. Rev. E* **84**, 011607 (2011).
23. Nguyen, V. D., Dang, M. T., Weber, B., Hu, Z. & Schall, P. Visualizing the structural solid–liquid transition at colloidal crystal/fluid interfaces. *Adv. Mater.* **23**, 2716–2720 (2011).
