# Supplementary material for: Imaging how thermal capillary waves and anisotropic interfacial stiffness shape nanoparticle supracrystals
Source: Nat Commun. 2020 Sep 11;11:4555. doi: 10.1038/s41467-020-18363-2 (PMC7486387; doi:10.1038/s41467-020-18363-2)
Supplement: Supplementary file 3 — Description of Additional Supplementary Files [file 41467_2020_18363_MOESM3_ESM.docx]

Description of Additional Supplementary Items

Title: Supplementary Movie 1.

Description: Videoclip illustrating the workflow of mapping the surface profiles from experimental setup and extracting physical parameters from surface profiles.

Title: Supplementary Movie 2.

Description: Synchronized movie showing the boundary of a growing supracrystal. From the original liquidphase TEM image (left), column positions are tracked, from which solid bond number for each column is calculated (middle, column positions color-coded by solid bond number). Surface profile is shown as the black line on the right, which is identified as the outmost layer of the supracrystal with solid bond number greater than or equal to 4 (gray dots). The movie is played at 13 frames per second (fps), 10 times real time. Scale bars: 200 nm. Dose rate: 3.7–14.8 e – ·Å– 2 ·s– 1 .

Title: Supplementary Movie 3.

Description: Synchronized movie showing the trajectory and displacement map of a growing supracrystal. On the left, we show the evolution of the trajectory map measured from the liquid-phase TEM movie. On the right, we show displacement maps of the columns close to the supracrystal–suspension interface (top, green) and inside supracrystal (bottom, blue), corresponding to the regions boxed in the left. The movie is played at 13 fps, 10 times real time. Scale bars: 200 nm.

Title: Supplementary Movie 4

Description:. Synchronized movie showing the surface roughness for stage 1 (top) and stage 2 (bottom). From the surface profiles (left), the temporal evolution of ℎ(𝑥,𝑡) (middle) and accumulated histogram (right) are calculated and presented. Scale bars: 200 nm.
