# Supplementary material for: Imaging how thermal capillary waves and anisotropic interfacial stiffness shape nanoparticle supracrystals
Source: Nat Commun. 2020 Sep 11;11:4555. doi: 10.1038/s41467-020-18363-2 (PMC7486387; doi:10.1038/s41467-020-18363-2)
Supplement: Supplementary file 2 — Peer Review File [file 41467_2020_18363_MOESM2_ESM.docx]

Peer Review Information

**Journal:** Nature Communications

**Manuscript title:** Imaging How Thermal Capillary Waves and Anisotropic Interfacial Stiffness Shape Nanoparticle Supracrystals

**Corresponding author name(s):** Prof Qian Chen

**Editorial notes:**

**none**

**Reviewer comments & decisions:**

**Reviewer comments, first version:**

Reviewers' comments:

Reviewer #1 (Remarks to the Author):

This manuscript describes the interfacial properties of supracrystals composed of triangular nanoprisms. The authors utilize in-situ liquid-phase TEM to image the growing interface of the supracrystal and characterize the interface using a capillary wave fit to determine the interfacial stiffness. The major finding is that the interfacial crystal facets of lower surface energy are favored. The experimental imaging via TEM is impressive, and the results and analysis are well done and interesting. The findings are in line with what is expected for interfacial properties of nanoscale crystal interfaces: the interfacial properties are dictated by a balance between thermal and surface energies.

The manuscript is focused on method development of interfacial characterization of one particular nanoparticle (NP) system: triangular prisms. The authors claim this method can be extended to elucidate the dynamic growth of various NP crystals, yet this needs to be realized. To elevate the novelty of the manuscript, it is recommended that the authors follow through on some of the proposed potential of the results by offering comparisons of the crystal growth for different NPs or illustrating new phenomena. The claim on page 15, line 307, "one can control the interfacial stiffness of facets by utilizing the existing toolkit of surface chemistry for NPs" is not realized in this manuscript.

Other comments:
1) The authors make a statement on page 9, line 185, regarding the supracrystal growth process in two stages. It is not clear what these two stages are referring to with regards to the supracrystal growth dynamics.

2) The interfacial profile is treated at a 1-D height function (page 11, line 223), yet the supracrystal interface is not 1D. What are the implications of using this 1D projection? Also, it is clear that there is anisotropy in the height of the interface in 3D based on the TEM images and supplemental videos. Although this may not change the finding of the facets of lowest energy based on the face-stacking preference, some discussion of what may be lost is using a 1D projection is needed.


Reviewer #2 (Remarks to the Author):

The authors describe the use of in-situ liquid cell TEM to study the assembly of triangular nanoparticles at solid liquid interfaces. The data is analyzed using in house matlab codes which enables the spatial location and orientation of the particles to be tracked with time. Using this data the authors the interfacial dynamics, interpret the data using capillary wave theory and determine factors such as the anisotropic interfacial stiffness.
As there is great interest in colloidal assembly of nanoparticles and the use of liquid cell TEM to directly measure dynamics, I believe this paper will be or high interested to a broad materials science community. The paper itself represents a significant advance in terms of our ability to measure interfacial dynamics based on the details and quantitative data analysis. The liquid cell TEM studies are very high quality, this is exceptional data.
As such I would recommend publication after the following minor comments have been addressed

1. How much is the ionic strength increased during the experiment?
2. Can the authors determine how can particles are in each column from the contrast?
3. Do the authors believe there is a minimum number of layer required to see these dynamics?
4. Can you authors comment on the illumination area they used for the experiments?
5. Although the authors have previously established that the imaging conditions are low dose with respect to the surface ligands and also to the final crystal structure, it is still the case that the electron beam will generate reactive species in the cell that diffuse away. Therefore LPTEM experiments are often considered to be a steady state situation rather than an equilibrium. As such it is interesting or even surprising that the particles behave according to CWT. I believe it would be useful for the field for the authors to specifically comment on why/how the equilibrium process can be observed under a chemical steady state condition.


Reviewer #3 (Remarks to the Author):

The authors demonstrate the ability to analyze of the crystallization of 1D stacks of gold nanoprisms in the context of capillary wave theory by tracking the assembly process at the single-particle level using liquid-phase TEM techniques. This allows for numerous structural and thermodynamic parameters of the system to be extracted and monitored over time, resulting in a better understanding of near-equilibrium crystallization processes occurring via nanometer-scale colloidal particles. Overall, I enjoyed reading the manuscript and felt that did an excellent job applying important concepts from the colloidal physics community to nanometer-scale systems. However, I am confused as to the target audience of this work and have some misgivings about its significance. It seems to me that the results are straightforward enough that a reader familiar with colloid physics will find them uninteresting while a reader familiar with nanoparticle assembly will have difficulty understanding the analysis. First, there is not a clear rationale for why investigating supracrystal interfaces in nanometer scale particles should be different or more interesting than using micron-sized colloids, which has been done countless times in the past. Indeed, the fact that capillary wave theory works so well to describe the system implies that the physics are scale-independent and the results are in keeping with the extensive previous literature on this topic. On the other hand, I felt as though the manuscript was not written in a manner that would facilitate the understanding of a reader that is not already familiar with capillary wave theory or this sort of analysis. Many of the detailed parameters extracted from the videographs are not explained well and it is assumed that the reader already knows something of their significance. Consequently, I am afraid that this paper will appeal to neither the nanoscience community nor the colloid physics community. In addition, I am having some difficulty in understanding the authors view on the significance of the work. There is mention of many other nanoscale systems that could be analyzed in the manner described here, all of which would be interesting but none of which would be straightforward to image and analyze, e.g. lipid bilayers. This feels like something of a red herring to me and I’d like to see a stronger motivation for why understanding this particular system of nanometer scale particles might lead to new physics, a new material, new properties, etc... Finally, although I applaud the authors for the thoroughness of their analysis, at times I found it almost painfully detailed. There are figures in which virtually identical pieces of information are displayed in several different ways, making the process of reading and understanding the paper far more work than is really necessary. As a result, although I feel that this is really nice and interesting work, my first inclination is to suggest that it belongs in a more specialized journal, not one like Nature Communications that appeals to an extremely broad audience. However, I remain open to the possibility that the manuscript could be written in a way that addresses my concerns so I am happy to review a revised draft. I provide more specific comments and questions below:

• Isn’t the system investigated better described as a colloidal solid-gas interface, not a colloidal solid-liquid interface, as the authors have described it? Adjacent to the crystalline region there is no dense but poorly-ordered phase, only nanoparticle dispersed in a dilute solution. Many of the papers referenced by the authors that also use capillary wave theory to investigate colloidal interfaces indeed contain a solid-liquid interface, not a solid-gas interface. How does the fact that particles are not in a liquid state next to the crystal surface influence the analysis presented?
• I am somewhat concerned by the fact that the assembly process being observed in the liquid-cell TEM videos consists of numerous independent steps (since it is a hierarchical system) that are being ignored and simplified in the analysis. As I understand it, the dark objects in Figure 1c are not individual nanoparticles, but stacks of several dozen particles being viewed down their long axis. As a result, there are numerous single-particle crystallization steps necessary just to construct a single stack. So when stacks near the interface of the crystal are labeled as having a lower bond orientational order parameter or experience larger displacements, is this due to the behavior of a single stack moving as a whole cohesive unit, or is this better explained by dynamics occurring out-of-stack as a consequence of single-nanoparticle motions? I’m not sure that the imaging modality is really capable of distinguishing these differences and I wonder if ignoring the behavior of individual nanoparticles within a single stack is missing some of the more interesting dynamics of the system. Does the multi-step nature of the assembly process have any bearing on the capillary wave analysis provided in the manuscript?
• How is the value of Sc=0.175 determined as the threshold, above which bonds are considered “solid” in the calculation of the solid bond number? I can’t seem to find any reasoning for this value in the maintext or SI.
• In figure 2 the RMS roughness of the interface is described as being constant over time but in figure 3 the authors discuss how the RMS roughness changes over time. Is the difference here between the roughness of a single growing surface facet (constant RMS roughness over time) in figure 2 vs. the change in growing surface facet to one with lower energy (different RMS roughness over time) in figure 3? If so, this needs to be clarified in the writing.
• On page 14, the authors claim that discreteness and nonadditivity make modeling of nanoparticle systems difficult, and use this as a justification for the single-particle measurements presented here. Yet the paper demonstrates triumphantly that capillary wave theory is a fantastic framework for modeling the behavior of nanoparticle systems. It therefore seems as if the success of the model actually undercuts the necessity of doing such detailed measurements in the first place since the theory works so well.
• Since the authors make the point that their single-particle analysis and tracking is important for understanding the dynamics of the system, I believe they should be required to provide their code if the work is to be published in Nature Communications. The code is crucial to understanding and reproducing the work so including it seems to be consistent with the Editorial and Publishing policies listed on the Nat. Comm. website.

**Author rebuttal, first version:**

***Reviewer 1***

We appreciate the overall positive comments of Reviewer 1 who considered our “experimental imaging via TEM is impressive” and “the results and analysis are well done and interesting”. As the reviewer mentioned, our findings are in line with the established expectation of interfacial properties for nanoscale crystal. This consistency highlights the success of our imaging protocol in taking care of the complications from electron beam often existing in liquid-phase TEM studies. We thank the reviewer for the constructive comments, all of which we have addressed with new interaction calculations in Supplementary Fig. 2, new discussions and references in “Introduction” and “Discussion”, and new analysis in Supplementary Note 2 in the revised manuscript.

**Comment 1:** “*The authors claim this method can be extended to elucidate the dynamic growth of various NP crystals, yet this needs to be realized. To elevate the novelty of the manuscript, it is recommended that the authors follow through on some of the proposed potential of the results by offering comparisons of the crystal growth for different NPs or illustrating new phenomena. The claim on page 15, line 307, "one can control the interfacial stiffness of facets by utilizing the existing toolkit of surface chemistry for NPs" is not realized in this manuscript.*”

**Reply:** We thank the reviewer for the great suggestion to make our paper stronger! In the revised manuscript, we made the following changes to articulate the novelty and significance of the work and the opportunities of using the toolkit of surface chemistry for modulating the interfacial stiffness of facets.

1.	We completely rewrote the introduction section of our paper to emphasize the novelty and significance

of our work in the context of crystal growth,

“The shape and surface morphology of crystalline nanomaterials are of central importance to determine their properties and applications^1-4^. For example, surface roughness alone has been shown to restrict phonon transport below the Casimir limit in crystalline Si nanowires^3^, and to dictate cellular adhesion and proliferation on zirconia nanofilms^4^. When it comes to “supracrystals” assembled non-covalently from nanoparticles (instead of atoms), the crystal shape and exposed external facets can play a key role in governing their architecture-dependent properties^1,2,5^. For example, supracrystals composed of gold nanoparticles have been shown to exhibit photon–plasmon coupling for potential applications in lasing and quantum electrodynamics^2^, with their photonic modes encoded simply by the supracrystal shape. Numerous theoretical efforts have thus been focused on predicting the shape and surface morphology of crystals (i.e., crystal habit) developed during the growth process over the last few decades^6,7^. Regarding crystal shape, the Wulff construction rule is a prominent principle predicting the thermodynamically stable shape of a crystal by considering the collective effects of the surface energies of different facets of a crystal^7,8^. Extensions of the Wulff construction rule to Winterbottom and Summertop constructions further predict the crystal shapes when the crystal growth is confined by substrates^9^. Regarding surface morphology, a diversity of models on kinetic growth mode such as the Stranski–Krastanov, Frank–van der Merwe, and Volmer–Weber models weigh the balance of factors such as energy landscape of the crystal surface and surface diffusional dynamics, to predict whether building units attach layer-by-layer (relatively smooth) or as islands (relatively rough) to growing crystals^10,11^.

However, despite the broad interest and extensive theoretical models in crystal habit, achieving a high level of control over it at the nanoscale requires addressing a few fundamental gaps. For supracrystals, controlling their surface roughness relies on understanding how nanoparticles attach one by one onto the surface of a growing supracrystal during the self-assembly process in solution, which has been experimentally inaccessible due to the challenge to resolve the motions of individual nanoparticles in solutions at the nanometer resolution^12^. As a result, supracrystal growth dynamics have been largely indirectly inferred from either ex-situ real-space measurements based on electron microscopy images

of discrete stages of the growth process in dried samples^13,14^, or in-situ reciprocal space techniques like small angle X-ray scattering^15^. As to controlling supracrystal shapes, the encountered challenges are threefold. All the construction rules (e.g., Wulff, Winterbottom, Summertop) based on thermodynamic stability require precise surface energy values of different supracrystalline facets as the inputs, which have not been experimentally measured^8,9^. Instead, these values were estimated by a broken bond model assuming all the interparticle interactions are local, which can fail due to the long-range nature of most colloidal interactions at the nanoscale^16^ or when the building units are highly anisotropic in shape (e.g., rods, prisms, arrows) and render the interparticle interactions directional and non-identical to each other. Moreover, nanoparticles can experience high diffusion barriers compared to atoms that these theories were originally established for, so that they can easily fall into kinetically trapped states which deviate from thermodynamic predictions^17^. Thirdly, simulations of their growth behaviors need to consider the nanoscopic morphology and interaction profiles of the nanoparticles, and the mass and energy transport contributions—surface and volume diffusion, defect annealing, strain relaxation—all at once, which remains challenging and computationally expensive, especially in the absence of experimental data.”

2.	We added new discussions and literatures in the “Discussion” section on how to control the interfacial

stiffness using the toolkit of nanoparticle surface chemistries and beyond,

“Our capability to measure facet-dependent interfacial stiffness and our demonstration on their role in shaping the supracrystals can advance crystal design at the nanoscale. For example, one can control the interfacial stiffness and crystal habit by utilizing the toolkits of both intrinsic parameters of nanoparticle shape and surface chemistry^41,54-56^ as well as extrinsic parameters such as temperature, pH and ionic strength^57-59^. Previously reports have shown that changing the length of DNA ligands on the same gold nanoparticles has led to tunability in the lattice symmetry of the interior structure and exposed facets of the supracrystal^17,55,60^. In binary systems, a diversity of supracrystal shapes including the exotic diamond-like lattices has been achieved by introducing two ligand types, two differently sized nanoparticles, or two nanoparticle compositions^61^. Our approach can elucidate the fundamental parameter of interfacial stiffness in these systems and make the crystal design predictable... In-situ liquid-phase TEM is also compatible with introducing environmental triggers during the observation,^18,65^ to dynamically modify the surface roughness and interfacial stiffness of crystals as one sees the growth, which can lead to the emergence of a “modulation on demand” strategy.”

**Comment 2:** “*The authors make a statement on page 9, line 185, regarding the supracrystal growth process in two stages. It is not clear what these two stages are referring to with regards to the supracrystal growth dynamics.*”

**Reply:** We thank the reviewer for helping us clarify our work better. In the original manuscript, we defined the two stages in Fig. 3a and Supplementary Fig. 8 (Supplementary Fig. 9 in the revised manuscript) with main text discussions: “The temporal evolution of surface orientation 9 exhibits two stages (labeled in salmon and aquamarine), each of which follows its own Gaussian distribution.” and “9 is defined as the angle between the linear regression of the interfacial profile^45^ tracked in each TEM snapshot and a horizontal axis (Supplementary Note 9).”

In the revised manuscript, we made the two stages more clearly by adding the following three discussions:

1. Articulated the difference in surface orientation 9 as the signature differentiating the two stages: “The surface orientation 9 undergoes a 19% increase, starting from 9 = (36 ± 6)° for the first stage of ~100 s, and then shifting to stabilize at 9 = (49 ± 5)° for the second stage of ~50 s (Supplementary Fig. 9a).”
2. Discussed the relevance to growth dynamics: “The surface orientation dictates the exposed facets (as well as the growth direction) of the supracrystal, which in our experiment changes from a high index

(340) facet to a low index (210) facet, converging towards thermodynamically favored lower surface energy state.”

3.	We presented the full temporal evolution of RMS roughness in the modified Fig. 2g, whose change is another signature of the two-staged supracrystal growth besides that of the surface orientation: “Further analysis of root mean square (RMS) roughness versus time shows a two-stage evolution which we discuss more in detail later: RMS roughness stays constant at (104 ± 36) nm during the first ~100 s and stabilizes to a lower value of (80 ± 25) nm for the later ~50 s (Fig. 2g, Supplementary Note 8, Supplementary Fig. 9)”.

**Comment 3:** “*The interfacial profile is treated at a 1-D height function (page 11, line 223), yet the supracrystal interface is not 1D. What are the implications of using this 1D projection? Also, it is clear that there is anisotropy in the height of the interface in 3D based on the TEM images and supplemental videos. Although this may not change the finding of the facets of lowest energy based on the face-stacking preference, some discussion of what may be lost is using a 1D projection is needed.*”

**Reply:** We thank the reviewer for helping us clarify this important detail! In summary, using a 1-D height function is correct in our system for two reasons. First, the stacked prisms in a column move as a whole due to a huge face-to-face attraction (of a magnitude > 20 *k*B*T*); all the prisms composing one single column share the same centroid positions (*x, y*), which match with the projected centroid position of the column that we tracked. Second, the intercolumn interaction dictating the interfacial stiffness in our system is insensitive to the small variations of the column cross-section along the columns (i.e., “the anisotropy in the height of the interface in 3D” per the reviewer) as we detail below. In the revised manuscript, we added new calculations, one new Supplementary Fig. 2, one new Supplementary Note 2, and new discussions listed below:

1. Added new interaction calculation results and schematics (Supplementary Fig. 2) to rationalize the use of a 1-D height function in our system: The pairwise interaction calculation presented in Supplementary Fig. 2d shows that the face-to-face stacking of two adjacent prisms is most favored at a configuration where the centroid (*x, y*) positions of the two prisms are the same. The energy penalty for the two prisms to deviate from this common centroid by as small as 10 nm (~10% of the prism side length, our tracking error) is already 2 *k*B*T*, not favored when agitated only by thermal fluctuations. Supplementary Figs. 2a‒c provide schematics illustrating the conclusions above.
2. Added a new supplementary note (Supplementary Note 2) discussing the results above and the radially symmetric intercolumn interactions. As to the latter, previous computational work on the same system (Ref. 31) has shown that the intercolumn interaction is isotropic as if the columns are circular cylinders, where the small variations caused by random misalignment do not impact the intercolumn interaction.
3. Added new discussions in the “Results” section on the choice of 1-D height function, “An ionic strength increase to ~0.5 M screens the interprism electrostatic repulsion to induce the face-to-face stacking of the prisms into cylindrical columns standing vertical to the SiN*_x_* substrate^31^. As shown in our pairwise interprism interaction calculation (Supplementary Fig. 2), the constitute prisms in one column adopt the most stable configuration where all of them share the same (, ) centroid positions as the column. At this configuration, the net attraction energy is huge, of a magnitude larger than 20 *k*B*T*. The energy penalty for the two adjacent prisms to deviate from this common centroid by as small as 10 nm (~10% of the prism side length) is already 2 *k*B*T*, not favored when agitated only by thermal fluctuations (Supplementary Fig. 2d, Supplementary Note 2). Accordingly, each column can be treated as one cylindrical object fully described by its projected (, ) centroid coordinates in our interfacial fluctuation analysis. Variations of the cross-sectional shape of the column does not affect the analysis either because the intercolumn interactions are radially symmetric (Supplementary Note 2).”

4. Added new discussions in the “Discussion” section to point out how to extend this 1-D height function analysis to a 2-D height function analysis for more complicated supracrystal structures, “Broadly, complete 2D surface profiles for more complicated supracrystal structures can be possibly achieved by TEM advancements such as SINGLE^62^, ultrafast electron tomography^63^ and focus-based 3D imaging with z-slices^64^, which can be analyzed following the protocols presented here.”

***Reviewer 2***

Reviewer 2 considered our work “of high interest to a broad materials science community” and presents “a significant advance in terms of our ability to measure interfacial dynamics” with “very high quality” liquid-cell studies and with “exceptional data”. The reviewer recommended publication with minor comments, all of which we have addressed as detailed below. We thank the reviewer for making our paper stronger!

**Comment 1:** “*How much is the ionic strength increased during the experiment?*”

**Reply:** We thank the reviewer for pointing out this important detail. In the revised manuscript, we added in the main text, “An ionic strength increase to ~0.5 M screens the interprism electrostatic repulsion to induce the face-to-face stacking of the prisms into cylindrical columns standing vertical to the SiN*_x_* substrate^31^.”

**Comment 2:** “*Can the authors determine how many particles are in each column from the contrast?*”

**Reply:** Sure! In the revised manuscript, we added in the main text, “The prisms in the columns are estimated to be 20*–*30, resulting in a circularly shaped projection of the columns due to sufficient orientational randomness of the prisms^31^” and Supplementary Note 2 to explain this estimation.

**Comment 3:** “*Do the authors believe there is a minimum number of layer required to see these dynamics?*”

**Reply:** We thank the reviewer for this thoughtful question. As the reviewer pointed out, there is a minimum number of prism layers (i.e., number of prisms in a column) needed for the hexagonal supracrystal to grow, although a single layer of prisms has enough TEM contrast for the dynamics to be observed. If the number of prisms inside one column is too few (e.g., smaller than 15, based on simulation estimation in Ref. 31), the column does not exhibit an effectively circular cross-section, resulting in a kinetically trapped assembly. In the revised manuscript, we added in the main text, “The prisms in the columns are estimated to be 20*–* 30, resulting in a circularly shaped projection of the columns due to sufficient orientational randomness of the prisms^31^.”

**Comment 4:** “*Can you authors comment on the illumination area they used for the experiments?*”

**Reply:** Sure! In the revised manuscript, we added the following details in the “Methods” section, “The illumination area is intentionally kept larger than the fluorescent screen of the TEM (~35 cm).” and “on a JEOL 2100 Cryo TEM with a spot size 3”. We also added discussions in the main text on the reason of our choice in the illumination area, “The electron beam is sufficiently spread out to minimize artefacts from the boundary and nonuniformity of the illumination area on the supracrystal growth and to achieve sufficiently low dose rates.”

**Comment 5:** “*Although the authors have previously established that the imaging conditions are low dose with respect to the surface ligands and also to the final crystal structure, it is still the case that the electron beam will generate reactive species in the cell that diffuse away. Therefore LPTEM experiments are often considered to be a steady state situation rather than an equilibrium. As such it is interesting or even surprising that the particles behave according to CWT. I believe it would be useful for the field for the authors to specifically comment on why/how the equilibrium process can be observed under a chemical steady state condition.*”

**Reply:** We thank the reviewer for the great comments to make our work more impactful for the field! In the revised manuscript, we now added the following in the main text,

“It is noteworthy that the radiolysis products generated during liquid-phase TEM imaging reach at steady concentrations within seconds^32,44^. At our low dose rates, these products do not react with nanoparticles but lead to an effective ionic strength increase that screens interprism electrostatic interaction, which triggers supracrystal growth^31,32,45^.”

***Reviewer 3***

Reviewer 3 appreciated the novelty and high-quality of our paper by commenting that the reviewer “enjoyed reading the manuscript and felt that it did an excellent job applying important concepts from the colloidal physics community to nanometer-scale systems,” and that our approach “allows for numerous structure and thermodynamic parameters of the systems to be extracted and monitored over time, resulting in a better understanding of near-equilibrium crystallization process occurring via nanometer-scale colloidal particles”. The reviewer also pointed out the importance of motivating the work better, especially on the significance of demonstrating the applicability of capillary wave theory (CWT) to the nanoscale, and the direct impact of our work on the field of nanoparticle supracrystals. We thank the reviewer for the greatly constructive and thorough comments, all of which we have addressed as detailed below in our point-to-point response.

As highlights of our changes, in the revised manuscript, we have (i) completely rewritten the introduction and did major revisions on the “Results” and “Discussion” sections to articulate the novelty and significance of our work and to clarify some experimental and analysis details; (ii) made an animation to vividly present our analysis workflow to the nanoparticle community (Supplementary Movie 1), and (iii) updated two of the four main figures and added 2 more supplementary figures and 1 supplementary note to help the readers who are not familiar with CWT or our analysis workflow understand our work better.

**Comment 1 on motivation of the work:** *“There is not a clear rationale for why investigating supracrystal interfaces in nanometer scale particles should be different or more interesting than using micron-sized colloids, which has been done countless times in the past. Indeed, the fact that capillary wave theory works so well to describe the system implies that the physics are scale-independent and the results are in keeping with the extensive previous literature on this topic,”* and “*having some difficulty in understanding the authors’ view on the significance of the work. There is mention of many other nanoscale systems that could be analyzed in the manner described here, all of which would be interesting but none of which would be straightforward to image and analyze, e.g. lipid bilayers. This feels like something of a red herring to me and I’d like to see a stronger motivation for why understanding this particular system of nanometer scale particles might lead to new physics, a new material, new properties, etc.”*

“*On page 14, the authors claim that discreteness and nonadditivity make modeling of nanoparticle systems difficult, and use this as a justification for the single-particle measurements presented here. Yet the paper demonstrates triumphantly that capillary wave theory is a fantastic framework for modeling the behavior of nanoparticle systems. It therefore seems as if the success of the model actually undercuts the necessity of doing such detailed measurements in the first place since the theory works so well.*”

**Reply:** We sincerely thank the reviewer for helping us make our paper stronger and more impactful to a broader readership! **First**, as the reviewer pointed out that our work is the first-time demonstration of CWT to a nanoscopic system suspended in solution. We hope to highlight that this applicability alone is a hallmark for the liquid-phase TEM community. This community is undergoing fast developments and has generated beautiful TEM movies on dynamics previously inaccessible. At the same time, this community is also at a crosspoint that many of the observations are self-contained and challenging to be connected with other communities due to complications from the imaging electron beam, substrate‒sample interaction and geometrically-confined, hindered diffusions. Therefore our work, together with the sample preparation and

imaging/analysis protocols we detailed in this paper, serves as a potent demonstration that the complications specific to liquid-phase TEM can be minimized and the results can be matched with theoretical predictions. We foresee our methods can be followed and used to apply other parts of the framework of colloidal physics (e.g., phase diagram mapping, grain boundary migration) to nanoparticles (or broadly nanosized entities), which has been long missing because colloidal physics rely on the real-space and real-time imaging of single particle level dynamics. **Second**, as the reviewer has also pointed out, CWT measures a myriad of useful physical parameters without requiring priori knowledge on materials-specific properties of the system (e.g., size, interaction profile of the building blocks). The only input needed are the directly imaged real-space and real-time movies—a perfect and timely match with liquid-phase TEM imaging. Although it remains debating in theory and simulation on how to model nanoscale colloidal interactions appropriately, CWT builds only on the assumption of equilibrium (or near-equilibrium) and works regardless of the details of the interaction profile (e.g., short-ranged vs long-ranged, additive vs non-additive). As we have now articulated a lot better in the revised manuscript, these parameters we measured under the framework of CWT fill a few key knowledge gaps in crystal growth and design at the nanoscale.

In the revised manuscript, we completely **rewrote** the introduction and “Discussion” sections as follows to clarify the significance and motivation of our work:

1.	For the introduction: we highlight the significance of the consistence of CWT, and the importance of

the parameters such as surface roughness, growth kinetics (i.e., crystal habit) and interfacial stiffness,

“The shape and surface morphology of crystalline nanomaterials are of central importance to determine their properties and applications^1-4^. For example, surface roughness alone has been shown to restrict phonon transport below the Casimir limit in crystalline Si nanowires^3^, and to dictate cellular adhesion and proliferation on zirconia nanofilms^4^. When it comes to “supracrystals” assembled non-covalently from nanoparticles (instead of atoms), the crystal shape and exposed external facets can play a key role in governing their architecture-dependent properties^1,2,5^. For example, supracrystals composed of gold nanoparticles have been shown to exhibit photon–plasmon coupling for potential applications in lasing and quantum electrodynamics^2^, with their photonic modes encoded simply by the supracrystal shape. Numerous theoretical efforts have thus been focused on predicting the shape and surface morphology of crystals (i.e., crystal habit) developed during the growth process over the last few decades^6,7^. Regarding crystal shape, the Wulff construction rule is a prominent principle predicting the thermodynamically stable shape of a crystal by considering the collective effects of the surface energies of different facets of a crystal^7,8^. Extensions of the Wulff construction rule to Winterbottom and Summertop constructions further predict the crystal shapes when the crystal growth is confined by substrates^9^. Regarding surface morphology, a diversity of models on kinetic growth mode such as the Stranski–Krastanov, Frank–van der Merwe, and Volmer–Weber models weigh the balance of factors such as energy landscape of the crystal surface and surface diffusional dynamics, to predict whether building units attach layer-by-layer (relatively smooth) or as islands (relatively rough) to growing crystals^10,11^.

However, despite the broad interest and extensive theoretical models in crystal habit, achieving a high level of control over it at the nanoscale requires addressing a few fundamental gaps. For supracrystals, controlling their surface roughness relies on understanding how nanoparticles attach one by one onto the surface of a growing supracrystal during the self-assembly process in solution, which has been experimentally inaccessible due to the challenge to resolve the motions of individual nanoparticles in solutions at the nanometer resolution^12^. As a result, supracrystal growth dynamics have been largely indirectly inferred from either ex-situ real-space measurements based on electron microscopy images of discrete stages of the growth process in dried samples^13,14^, or in-situ reciprocal space techniques like small angle X-ray scattering^15^. As to controlling supracrystal shapes, the encountered challenges are threefold. All the construction rules (e.g., Wulff, Winterbottom, Summertop) based on thermodynamic

stability require precise surface energy values of different supracrystalline facets as the inputs, which have not been experimentally measured^8,9^. Instead, these values were estimated by a broken bond model assuming all the interparticle interactions are local, which can fail due to the long-range nature of most colloidal interactions at the nanoscale^16^ or when the building units are highly anisotropic in shape (e.g., rods, prisms, arrows) and render the interparticle interactions directional and non-identical to each other. Moreover, nanoparticles can experience high diffusion barriers compared to atoms that these theories were originally established for, so that they can easily fall into kinetically trapped states which deviate from thermodynamic predictions^17^. Thirdly, simulations of their growth behaviors need to consider the nanoscopic morphology and interaction profiles of the nanoparticles, and the mass and energy transport contributions—surface and volume diffusion, defect annealing, strain relaxation—all at once, which remains challenging and computationally expensive, especially in the absence of experimental data.

Here we utilize a recently developed technique of liquid-phase transmission electron microscopy (TEM) to experimentally map for the first time the kinetic and thermodynamic parameters key to crystal growth at the nanoscale, including the kinetic growth profile, facet-dependent interfacial stiffness (the sum of surface energy and its second derivative), surface roughness, and interfacial mobility. Mapping of these otherwise inaccessible parameters is made possible by combining a capillary wave theory (CWT) and the capability to directly image a fluctuating surface of a growing crystal at the nanoscale using liquid-phase TEM. Liquid-phase TEM can seal liquid samples against the high vacuum of TEM and capture the dynamics of nanosized entities at the nanometer resolution^18,19^. In this work, we use low-dose liquid-phase TEM imaging to study the shaping process of nanoparticle supracrystals during post-nucleation growth. Aided by single particle tracking, we find that the spatiotemporal fluctuations of the surface profiles of a growing supracrystal match quantitatively with CWT (Supplementary Movie 1), which has been prevalently used for equilibrated interfaces in micron-sized colloid^20,21^ and atomic systems^22-26.^ This match is demonstrated for the first time on the single nanoparticle level—only accessible with liquid-phase TEM—showcasing the capability of our method to minimize long-standing complications from beam or substrate. From the liquid-phase TEM movies, we capture an intriguing shift in the exposed facets (from (340) to (210)) during the supracrystal growth. Following CWT, we are able to measure the corresponding interfacial stiffness, which pinpoints the energetic driving force for this shift towards lower surface energy facets, resulting in a smoother faceted morphology of the supracrystal. This real-space imaging and fluctuation analysis of a growing nanoparticle supracrystal provides guidance on encoding the shape and surface morphology of nanostructured materials from the building units and growth kinetics. Our workflow can be potentially extended to other phenomena governed by nanoscale interfacial fluctuations, from substrate guided crystal growth^17^, dendrite formation^27^, fusion of lipid vehicles^28^, to cellular compartmentalization^29^.”

2.	In the “Discussion” section, we substantially elaborated the impact of our work on future nanomaterials

design:

“Our capability to measure facet-dependent interfacial stiffness and our demonstration on their role in shaping the supracrystals can advance crystal design at the nanoscale. For example, one can control the interfacial stiffness and crystal habit by utilizing the toolkits of both intrinsic parameters of nanoparticle shape and surface chemistry^41,54-56^ as well as extrinsic parameters such as temperature, pH and ionic strength^57-59^. Previously reports have shown that changing the length of DNA ligands on the same gold nanoparticles has led to tunability in the lattice symmetry of the interior structure and exposed facets of the supracrystal^17,55,60^. In binary systems, a diversity of supracrystal shapes including the exotic diamond-like lattices has been achieved by introducing two ligand types, two differently sized nanoparticles, or two nanoparticle compositions^61^. Our approach can elucidate the fundamental parameter of interfacial stiffness in these systems and make the crystal design predictable. Broadly, complete 2D surface profiles for more complicated supracrystal structures can be possibly achieved by TEM advancements such as SINGLE^62^, ultrafast electron tomography^63^ and focus-based 3D imaging

with z-slices^64^, which can be analyzed following the protocols presented here. In-situ liquid-phase TEM is also compatible with introducing environmental triggers during the observation,^18,65^ to dynamically modify the surface roughness and interfacial stiffness of crystals as one sees the growth, which can lead to the emergence of a “modulation on demand” strategy.”

**Comment 2 on accessibility to non-colloidal physics readers:** *“On the other hand, I felt as though the manuscript was not written in a manner that would facilitate the understanding of a reader that is not already familiar with capillary wave theory or this sort of analysis. Many of the detailed parameters extracted from the videographs are not explained well and it is assumed that the reader already knows something of their significance.” And “There are figures in which virtually identical pieces of information are displayed in several different ways, making the process of reading and understanding the paper far more work than is really necessary.”*

**Reply:** We thank the reviewer for making our paper more broadly accessible! In the revised manuscript, in addition to rewriting the “Introduction” and “Discussion” sections as discussed in our reply to Comment 1, we have made the following changes to further help a reader that is not familiar with CWT understand our analysis workflow better:

1. We made an animation to illustrate the workflow and concepts presented in our paper (Supplementary Movie 1): In this animation, we integrated schematics and experimental data, covering details from the experimental setup of liquid-phase TEM, image analysis, CWT, to physical parameter extraction, in order to make our analysis and results more approachable to a broader community.
2. We updated Figures 2 and 3 to avoid potential misinterpretation of our data: We moved the original Figure 3d,e, which cover the technical details on the derivation of height profiles, into Supplementary Figure 10. We replaced the original Figure 2g with the full-time RMS *vs.* time profile (including two growth stages) to avoid confusion.
3. We added another one supplementary figure and one supplementary note: In the revised Supplementary Figure 2 and Supplementary Note 2, we added discussions on the motion and structure of single prisms in the surpracrystal, which serve as the underlying basis of the fluctuation analysis.
4. We thoroughly edited the “Results” section as highlighted in the revised manuscript to explain more vigorously the meaning and significance of each parameter as we presented it.

**Comment 3:** “*Isn’t the system investigated better described as a colloidal solid-gas interface, not a colloidal solid-liquid interface, as the authors have described it? Adjacent to the crystalline region there is no dense but poorly-ordered phase, only nanoparticle dispersed in a dilute solution. Many of the papers referenced by the authors that also use capillary wave theory to investigate colloidal interfaces indeed contain a solid-liquid interface, not a solid-gas interface. How does the fact that particles are not in a liquid state next to the crystal surface influence the analysis presented?*”

**Reply:** In our original manuscript, we motivated our work by examples of “solid-liquid” interfaces. For the reasons the reviewer has nicely presented, we referred to the interface as between the supracrystal and suspension. We made that choice because we were concerned that referring to the suspension as “gas” phase might cause confusion (because physically it is in liquid water) to the nanoparticle community. Thanks to the reviewer’s suggestions, in the revised manuscript, we now motivate our work directly on crystal growth (see our reply to Comment 1) and do not discuss “solid-liquid interface”. As a result, we have unified the terminology as either “supracrystal surface” or “interface between the supracrystal and the suspension” for clarity to a broader community.

We also would like to note that CWT considers the generic balance between thermally driven fluctuation and surface energy, independent of the states of the two phases interfaced. This theory has been applied to different interfacial systems, such as solid-liquid (*Proc. Natl. Acad. Sci. U.S.A.*, 106, 15198–15202, 2009), solid-solid (*Phys. Rev. X*, 8, 021045, 2018), liquid-gas (*Science*, 304, 847–850, 2004) and solid-gas (*Phys. Rev. Materials*, 2, 025602, 2018). Our work is the first demonstration and application of this theory at the single nanoparticle level in the context of supracrystal growth.

**Comment 4:** “*I am somewhat concerned by the fact that the assembly process being observed in the liquid-cell TEM videos consists of numerous independent steps (since it is a hierarchical system) that are being ignored and simplified in the analysis. As I understand it, the dark objects in Figure 1c are not individual nanoparticles, but stacks of several dozen particles being viewed down their long axis. As a result, there are numerous single-particle crystallization steps necessary just to construct a single stack. So when stacks near the interface of the crystal are labeled as having a lower bond orientational order parameter or experience larger displacements, is this due to the behavior of a single stack moving as a whole cohesive unit, or is this better explained by dynamics occurring out-of-stack as a consequence of single-nanoparticle motions? I’m not sure that the imaging modality is really capable of distinguishing these differences and I wonder if ignoring the behavior of individual nanoparticles within a single stack is missing some of the more interesting dynamics of the system. Does the multi-step nature of the assembly process have any bearing on the capillary wave analysis provided in the manuscript?*”

**Reply:** We thank the reviewer for helping us clarify this important detail. In this work we consider the post-nucleation crystal growth, at which stage a single stack of face-to-face positioned prisms indeed moving as a whole cohesive unit. This fact serves as the basis of our analysis to focus on the projections of the columns. The stacked prisms in a column move as a whole due to a huge face-to-face attraction (of a magnitude > 20 *k*B*T*); all the prisms composing one single column share the same centroid positions (*x, y*), which match with the projected centroid position of the column that we tracked and analyzed. We did have observed remarkable multi-step assembly in the system during the initial emergence of crystallites (i.e., nucleation), which we discussed in detail in Ref. 31. In the revised manuscript, we added new calculations, one new Supplementary Fig. 2, one new Supplementary Note 2, and new discussions listed below:

1. Added new interaction calculation results and schematics (Supplementary Fig. 2), and Supplementary Note 2 to rationalize a single stack of face-to-face positioned prisms indeed move as a whole cohesive unit: The pairwise interaction calculation presented in Supplementary Fig. 2d shows that the face-to-face stacking of two adjacent prisms is most favored at a configuration where the centroid (*x, y*) positions of the two prisms are the same. The energy penalty for the two prisms to deviate from this common centroid by as small as 10 nm (~10% of the prism side length, our tracking error) is already 2 *k*B*T*, not favored when agitated by thermal fluctuations. Supplementary Figs. 2a-c provide schematics illustrating the conclusions above. These new calculation results were discussed in Supplementary Note 2.
0. Added new discussions in the “Results” section on the choice of 1-D height function, “An ionic strength
   increase to ~0.5 M screens the interprism electrostatic repulsion to induce the face-to-face stacking of the prisms into cylindrical columns standing vertical to the SiN*_x_* substrate^31^. As shown in our pairwise interprism interaction calculation (Supplementary Fig. 2), the constitute prisms in one column adopt the most stable configuration where all of them share the same (, ) centroid positions as the column. At this configuration, the net attraction energy is huge, of a magnitude larger than 20 *k*B*T*. The energy penalty for the two adjacent prisms to deviate from this common centroid by as small as 10 nm (~10% of the prism side length) is already 2 *k*B*T*, not favored when agitated only by thermal fluctuations (Supplementary Fig. 2d, Supplementary Note 2). Accordingly, each column can be treated as one cylindrical object fully described by its projected (, ) centroid coordinates in our interfacial fluctuation analysis. Variations of the cross-sectional shape of the column does not affect the analysis either because the intercolumn interactions are radially symmetric (Supplementary Note 2).”

**Comment 5:** “*How is the value of Sc=0.175 determined as the threshold, above which bonds are considered “solid” in the calculation of the solid bond number? I can’t seem to find any reasoning for this value in the maintext or SI.*”

**Reply:** In the original manuscript, we described the determination of the threshold *S*c based on the bimodal distribution of *Sjk* in both the main text “The histogram of shows a characteristic of 0.175 (Fig. 2a,b)” and in the figure caption of Fig. 2b, “A histogram of the connectivity computed from Supplementary Movie 1, with c denoted with the black arrow.” In the revised manuscript, we further clarified it by adding one sentence in the main text, “The histogram of shows a characteristic _c_ of 0.175, chosen as the local minimum between the two peaks of the histogram (Fig. 2a,b).”

**Comment 6:** “*In figure 2 the RMS roughness of the interface is described as being constant over time but in figure 3 the authors discuss how the RMS roughness changes over time. Is the difference here between the roughness of a single growing surface facet (constant RMS roughness over time) in figure 2 vs. the change in growing surface facet to one with lower energy (different RMS roughness over time) in figure 3? If so, this needs to be clarified in the writing.*”

**Reply:** Thanks! As the reviewer correctly pointed out: the difference in Fig. 2 and Fig. 3 is that we only plotted RMS roughness of the first stage in Fig. 2, but considered full time span in Fig. 3. To eliminate the confusion, in the revised manuscript, we modified Fig. 2g to include the full evolution of RMS roughness.

**Comment 7:** “*Since the authors make the point that their single-particle analysis and tracking is important for understanding the dynamics of the system, I believe they should be required to provide their code if the work is to be published in Nature Communications. The code is crucial to understanding and reproducing the work so including it seems to be consistent with the Editorial and Publishing policies listed on the Nat. Comm. website.*”

**Reply:** We thank the reviewer for appreciating the importance of our code package to the community! We have now packaged the codes and released it on GitHub:

<https://github.com/chenlabUIUC/particle-identification>

**Reviewer comments, second version:**

Reviewer #1 (Remarks to the Author):

The authors have adequately addressed the previous concerns and comments regarding their manuscript. The results are interesting and the application of fast TEM imaging that can assist with a better understanding of dynamics at the nanoscale. They have clarified the sections involving the description of crystal phenomena. Adding a specific link for their codes will be valuable to those in the TEM and particle imaging community.


Reviewer #2 (Remarks to the Author):

I commend the authors on the revised version of the manuscript. I have no further comments and recommend publication as is


Reviewer #3 (Remarks to the Author):

The authors have written a detailed response and have addressed all of my comments. This is really beautiful work and I hope to see it published in Nature Communications shortly.

**Author rebuttal, second version:**

**Reviewer comments, third version:**

**Author rebuttal, third version:**
